# An ethnobotanical study of medicinal plants used to treat skin diseases in northern Pakistan

**DOI:** 10.1186/s12906-019-2605-6

**Published:** 2019-08-13

**Authors:** Khafsa Malik, Mushtaq Ahmad, Muhammad Zafar, Riaz Ullah, Hafiz Majid Mahmood, Bushra Parveen, Neelam Rashid, Shazia Sultana, Syed Nasar Shah, Lubna

**Affiliations:** 1 Department of Botany, Rawalpindi Women University, Rawalpindi, Pakistan; 2 Government Postgraduate College Women, UOG (University of Gujrat) Sub- Campus Rawalpindi, Rawalpindi, Pakistan; 3 Department of Plant Sciences, Quaid-i-Azam University, Islamabad, 45320 Pakistan; 4 Center for Natural Products Lab, Chengdu Institute of Biology, Sichuan, China; 5 Medicinal, Aromatic and Poisonous plant Research Centre (MAPRC), College of Pharmacy, King Saud University, box 2457, Riyadh, PO 11451 Saudi Arabia; 6 Department of Pharmacognosy, College of Pharmacy, King Saud University, box 2457, Riyadh, PO 11451 Saudi Arabia

**Keywords:** Skin diseases, Medicinal plant, Northern Pakistan, Traditional, Ethnomedicines

## Abstract

**Background:**

Skin diseases are a major health concern especially in association with human immune deficiency syndrome and acquired an immune deficiency. The aim of this study was to document the ethnomedicinal information of plants used to treat skin diseases in Northern Pakistan. This is the first quantitative ethnobotanical study of therapeutic herbs utilized by the indigenous people of Northern Pakistan for skin diseases.

**Methods:**

Interviews were taken to obtain information from 180 participants. Quantitative methods including fidelity level (FL), Frequency of citation (FC), Use-value (UV), Jaccard indices (JI), Family importance value (FIV), Relative frequency of citation (RFC) and Chi-square test were applied. Medicinal plants uses are also compared with 50 national and international publications.

**Results:**

In this study, we recorded 106 plant species belonged to 56 floral families for treatment of skin ailments. The dominant life form reported was herb while the preferred method of utilization was powder, along with leaf as the most used plant part. RFC ranges from 0.07 to 0.25% whereas the highest FIV was recorded for family Pteridaceae. FL values range from 36.8 to 100%. The study reported 88% of new plant reports for the treatment of skin diseases.

**Conclusion:**

The present study revealed the importance of several plants used to treat skin diseases by the local communities of Northern Pakistan. The available literature supported the evidence of plant dermatological properties. Plants having high UV and RFC can be considered for further scientific analysis. There is dire need to create awareness among local, government and scientific communities for the preservation of medicinal species and ethnomedicinal knowledge in Northern Pakistan.

**Electronic supplementary material:**

The online version of this article (10.1186/s12906-019-2605-6) contains supplementary material, which is available to authorized users.

## Background

Skin diseases present a major health concern worldwide [1]. Skin problems significantly affect the quality of health and difficult to treat due to persistence [2]. The skin is an external organ covering the body and serves many important functions including percutaneous absorption, organ protection, fluid preservation, body shape maintenance, temperature regulation and eliminating toxins from the body by sweat excretion [1]. The etiology of skin diseases display a close connection between an individual’s health and socio-cultural environment [3]. Skin diseases affect people of all age groups and gender [4]. Skin ailments or infectious dermatological dermatological diseases are particularly present in tropical areas of Globe [5]. Skin diseases constitute about 34% of all the ailments and supposed to be the most common disease among rural people [6]. Skin diseases have gained attention in recent years due to the association with AIDS/HIV. Greater than 90% of infectious persons of HIV developed mucosal and skin problems at certain phase of disease [1]. Skin ailments such as boils, itching, ringworm, skin disorders, leprosy, wound, dermatitis, eczema, scabies, skin allergy swelling and psoriasis are caused by a variety of microorganisms [7]. In previous reports, it was found that wound healing, eczema, dermatitis, fungal diseases, pyoderma, scabies, and skin allergies are the largest group of skin diseases that occur in most of the countries. Most of the plants used for treating skin disorders possibly have other additional properties like anti-inflammatory, anti-microbial, anti-viral, cicatrizant, hemostatic, analgesic effects that require pharmacological confirmation [8]. In literature, various plants have been reported to be used against skin infections like wound healing, scabies, swellings, boils, etc. [9–16].

In Pakistan, the number of patients suffering from skin diseases increases every year. The majority suffer from psoriasis, followed by pigment disorder, eczema, urticaria and fungal infection [17]. Climatic conditions like hot and humid weather intensify the prevalence of skin disorders. Although the mortality for skin infection is relatively low, the infection affects the quality of life. Modern skin therapies depend on the cause of the ailment. A skin disease caused by fungal and bacterial infection is medicated using antibiotics such as tolnaftate, clotrimazole, and gentamicin. It is believed that modern therapies have many disadvantages like antibiotic resistance, allergic and adverse reactions in some patients [18]. Modern medicines are very expensive with costly treatments so an alternative approach such as herbal medication in practiced.

Ethno-medicinal studies showed that herbal medicine is an alternative therapy for treatment and control of skin ailments [19]. Herbal anti-skin medicines have many useful properties including low side effects and cost treatment with high significant efficacy [20, 21]. Medicinal flora have shown a pivotal part in management of dermatological conditions [11, 22], particularly communities in developing countries local communities depend on traditional medicine for their health care [23]. The World Health Organization has a deep interest in the documentation of medicinal plant knowledge from from different areas of globe [24]. Currently, the Ministry of Public Health of Pakistan is promoting the usage of therapeutic herbs in health maintenance system [25].

In Pakistan, few previous reports exist the usage of therapeutic flora in skin care [1]. Therapeutic flora usage for treatment of skin ailments are documented in the literature [26], but, no specific study exists treatment of skin diseases. Various medicinal plants are also reported worldwide usage for the cure of skin disorders [7, 27–30]. The ethnobotanical literature on medicinal usage of flora for various ailments in Pakistan were mentioned in literature [31–37], but no systematic ethnomedicinal study has specifically focused on skin problems in the tribal areas of Northern Pakistan.

The objective of this research work is to document and examine the diversity of therapeutic flora used for treating the skin diseases in Northern Pakistan. This research will facilitate future scientific authentication through antimicrobial, pharmacological and phytochemical studies.

## Methods

### Description of study area

**Fig. 1:**
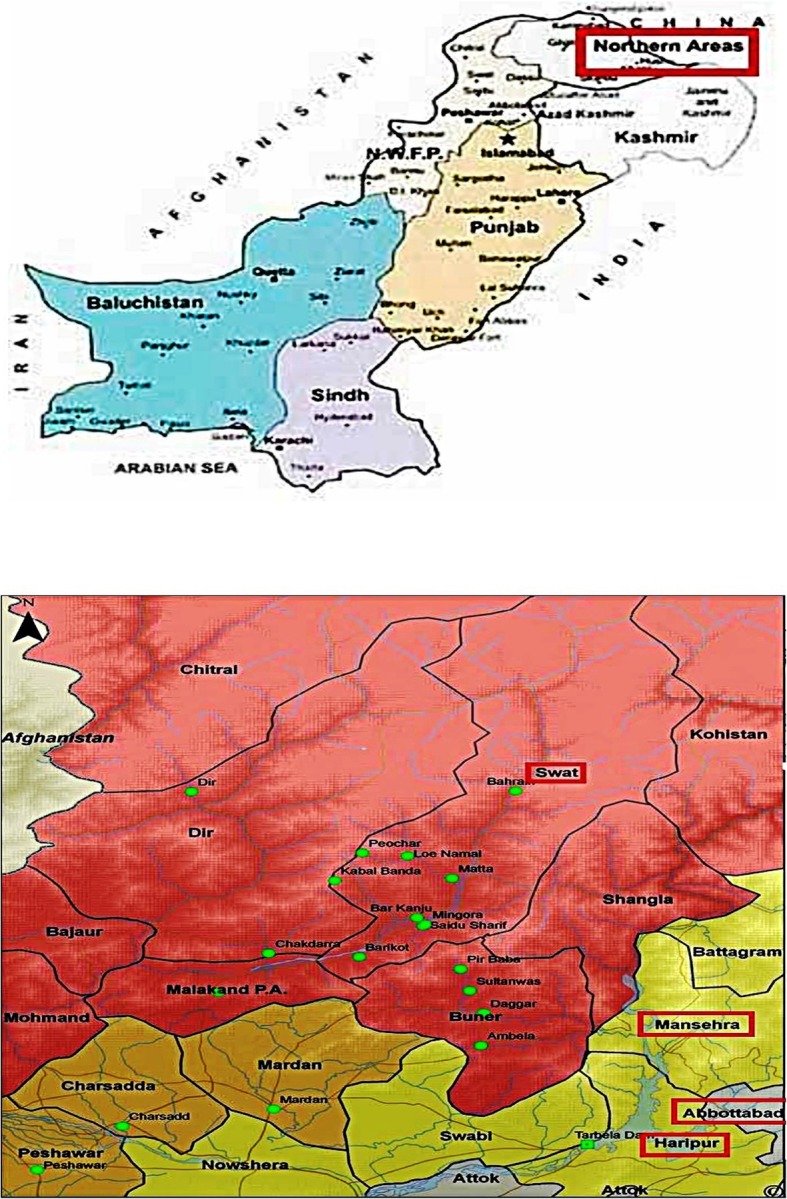
Map of the study area -Northern Pakistan (www.globalsecurity.org)

Northern Pakistan is home to the world's largest peaks and high mountain ranges i.e., Karakorum, Alai Ranges, Kunlun, Hindukush and Tien Shan [38]. Its topography differs from rock parts in North to green plains and forest in South. These areas are rich in floral variation of therapeutic plant species [39]. This area includes Hazara division, Swat valley, Mansehra, Kaghan and some tribal areas of Northern parts (Fig.1). The area is located at 72^°^35’to - 73_°_31′E and 33^°^50′-to 34^°^23′ N. The province borders Afghanistan to North Western side, Kashmir to East Punjab Islamabad capital territory to East and FATA to South. The average temperature recorded in the past was minimum in January as 1.7 °C while the mean maximum was 32.41 °C in June [40]. The average annual rainfall is about 1125 mm. The major tribes residing in the area include Khattak, Yusufzai, Marwat, Shinwari, Afridi, Orakzai, Mahsud, Mohmand, Abbassies, Wazir, Tareen, Mashwani, Jadoon, Tanolis, Awans, Sardars, Sheikhs and Qureshi [1]. Northern Pakistan is a hilly area and the cultivated land is not enough for sustenance [41]. Medicinal plant collection and other non-timber forest products provide an additional source of income (12%), while daily salaries and wages constitute 20%, transmittals from other areas of Pakistan and overseas (17%), and other occupations (10%) [41]. About 80% population in Pakistan is rural households and has easy access to medicinal plants.

### Ethical compliance

The present study was carefully designed with strict compliance of bio-ethics and approved by the Institutional Bioethics Committee (IBC) of Quaid-i-Azam University, Islamabad, Pakistan under the approval No PT-5695. The rules for plant collection and identification were followed according to National Biodiversity Action Plan as per the guidelines of Herbarium of Pakistan (ISL), Quaid-i-Azam University, Islamabad, Pakistan. Prior to data collection, a brief group discussion was held with the participants for agreement, to tell the objectives of research and to guarantee the safety of indigenous knowledge. These practices clear the aim of research and develop confidence in participants so they give reliable knowledge without any hesitation. Initially, 200 participants were selected of them were but among them, 20 were hesitant in providing knowledge leaving a total of 180 participants for data collection. While data documentation, all participants were contacted 3 times for the authentication of the knowledge given by informants. Any deviance of the informants idea from authentic knowledge given, the information was excluded and regarded inapplicable. The data quality was ensured through proper training of data collectors, pointing out missing information, duplication of the material, and careful analysis. The data quality was ensured through proper training of data collectors, pointing out missing information, duplication of the material, and careful analysis. The few plants in the MS are listed on the IUCN red list such as *Taxus wallichiana* (plant #104) is endangered*, Colubrina oppositifolia* (#84) is critical, *Aconitum chasmanthum* (#79) is critical and *Plantago lanceolata* (#69) is vulnerable. All plants listed in this study are authorized by the biodiversity action plan and duly authenticate by ethical committee of Quaid-i-Azam University, Islamabad and then included in the MS. The native communities of the area have knowledge about sustainable use of these plants and use of these plants with care (criteria of IUCN) so that they don’t get vanished and are save for next generation.

### Field study and data collection

This research work focused on the use of traditional plant resources with specific reference to the treatment of skin ailments. Fieldwork was performed between April 2015 to August 2015. Collectively, 180 participants were interviewed after receiving their prior informed consent. Data was collected from native indigenous health practitioners (THPs) and local participants (female and males of altered groups of age, experiences and education levels). During field surveys, face to face interviews and semi-structured interviews were also conducted. Guided field walks were also conducted [42]. The questionnaire used for data collection includes two parts, (i) part dealing with the demographic data of participants, and (ii) part focusing on information about plants´ local name, mode of administration, preparation and part of the plant used against skin diseases. Documentation of data while field survey was evaluated and organized by usage of quantitative analysis. In addition, data was compared with previously published research articles on ethnomedicinal uses of plants to validate the plants with higher medicinal values for skin diseases.

### Collection identification and preservation

In the current study, therapeutic flora documented by participants was identified by their common names [43]. The plant specimens were further authenticated by a Plant Taxonomist, Professor Mir Ajab Khan (Ph.D. in Plant Systematics) at the Herbarium of Pakistan (ISL), QAU Islamabad, Pakistan. All the plants species were further authenticated through available literature [42] and compared  with herbarium specimens. In addition, some plants used by the local healers were photographed. Voucher plant specimens were collected in duplicate. Herbarium specimens were deposited in Herbarium of Pakistan (ISL, Registered at Index Herbarium http://sweetgum.nybg.org/science/ih/) and voucher specimens are presented in.

### Quantitative data analysis

#### Use value (UV)

Use value is calculated to assess all probable usage of plant species. UV of plants gives a quantitative analysis for plant citation. UV tells the relative importance of plant flora recognized locally. UV was analysed according to [44].

$$ \mathrm{UV}=\mathrm{u}/\mathrm{N} $$

Where u is the total participants stating various uses of a plant and N is whole number of participants. UV is usually (1) if the number of usages is greater, and (0) if the usage report for plants species is less. UV not deliver data on multiple or single usage of plant flora is considerably low. UV does not deliver any data on the single or multiple uses of plant species.

#### Frequency of citation (FC) and relative frequency of citation (RFC*)*

FC is used for evaluating the most prefered plants or more used plant species. RFC was analysed to intricate the knowledge of traditional flora about usage of therapeutic flora in the study site.

$$ \mathrm{RFC}=\mathrm{FC}/\mathrm{N}\ \left(0<\mathrm{RFC}<1\right) $$

Where RFC is denoted by relative frequency citation, FC (Frequency of Citation) is the number of participants who stated the plant flora and N is whole number of informants [34].

#### Fidelity level (FL)

To analyse most prefered plant usage for the cure of a specific disease, we used (FL) index adopted by [37]. FFL indicates the importance of one species over other, to cure specific diseases. Fidelity level shows the percentage of participants who reported the use of specific plant species for a particular disease (Skin disease).

$$ \mathrm{FL}\ \left(\%\right)={\mathrm{N}}_{\mathrm{P}}/\mathrm{N}\times 100 $$

Where, Np is the number of participants that declare the usage of species for definite disease, and N is total participants that use plants as a medicines for the treatment of any given ailment [45].

#### Jaccard index (JI)

Jaccard index (JI) is evaluated by comparison of formarly published studies from local, regional and global level by analysing the percentage of cited plant species and medicinal usage, by using the following formula:

$$ \mathrm{JI}=\mathrm{c}\ \mathrm{multiply}\ 100/\mathrm{a}+\mathrm{b}-\mathrm{c} $$

where “a” is the number of species of area A, “b” is number of species of area B, and “c” is number of species common in A and B [46].

#### Chi-square test

The knowledge of medicinal species distributed between male and female participants between two age categories (36–46 and > 60 years of age) was comparatively analyzed by using Chi-square.

## Results

### Socio-demographic characteristics of participants

Collectively 180 participants were selected from several regions of Northern Pakistan. The majority of professional healers were males (61%). Based on age, the participants were divided into five groups (36–46 (11%), 47–57 (19%), 58–68 (24%), 69–79 (34%) and above 80 years (12%). Participants constitute 24 students, 41 herbalists, 32 physicians, 12 retirees, 46 housewives, 12 professionals, and 13 others. A large number (44%) of local healers also used allopathic medicines. Regarding education, 30% of the participants were illiterate, 35% of the traditional healers had attended primary school, 18% secondary education level, 9% tertiary education and only 8% of participants had attended universities. The majority of professional healers (43%) in the study area were married, followed by single (37%), widowed (16%) and 4% divorced Most of the participants were living in rural areas (88%) and only 12% living in urban areas (Table 1).

**Table 1:** Demographic data of participants

Parameters		Participants (N)	N (%)
Gender	Female	70	39
	Male	110	61
Age	36–46	20	11
	47–57	35	19
	57–67	43	24
	68–78	62	34
	80>	20	12
Education	No formal education	55	30
	Primary	63	35
	Secondary	32	18
	Tertiary	16	9
	Others	14	8
Collaboration with modern medicine	Collaborative	80	44
	Non collaborative	100	56
Occupation	Student	24	13
	Herbalists	41	23
	Physician	32	18
	Retired	12	7
	Housewife	46	25
	Professional	12	7
	Others	13	7
Residence	Urban	22	12
	Rural	158	88
Marital status	Single	66	37
	Married	78	43
	Widowed	29	16
	Divorced	7	4

### Diversity of medicinal plants used

**Fig. 2:**
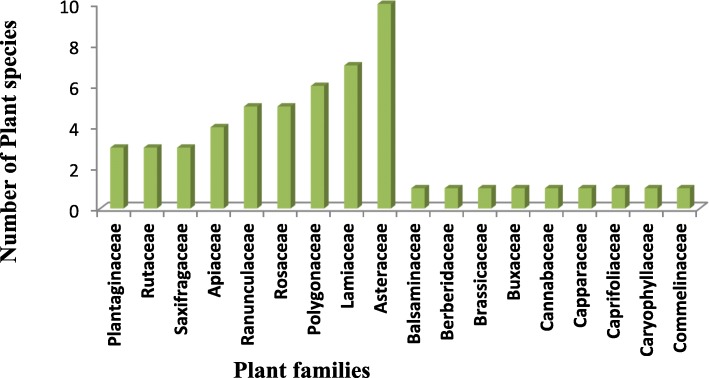
Dominant families of medicinal plants utilized for skin disorders in Northern Pakistan

Therapeutic flora, used to cure skin diseases in Northern Pakistan are documented in Table 2. The study reported 106 medicinal plant species. The main growth habit of the plant flora was herbs 62%, followed by shrubs (20%) and trees (18%). The plants belonged to 56 families. Asteraceae (10 species) and Lamiaceae (7 species) represent the most dominant family in this study site (Fig. 2). The other important families in the study included Polygonaceae (6 species), then Ranunculaceae and Rosaceae (5 species each). The least species (1%) were observed in 37 families (Fig. 2).

**Table 2:** Medicinal plants used for skin diseases in Northern Pakistan

Family / Scientific name / coll. #	Vernacular Name	Habit	Plant Part used	Mode of utilization	Disease treated	Preparation	FC	RFC	UV	FL	Comparison
Acanthaceae *Justicia adhatoda* L. LI 58	Behkar	Shrub	Leaf	Decoction, powder	Wound healing	Leaf are directly applied on wounds	23	0.13	0.043	73.91	1 □, 2 □, 3 ■, 4 □, 5 ■, 6 □, 7 □, 8 □, 9 □, 10 □, 11 □, 12 □, 13 □, 14 □, 15 □, 16 □, 17 □, 18 □, 19□, 20 □, 21□, 22 □, 23 □, 24 □, 25 □, 26 □, 27 □, 28 □, 29 □, 30 □, 31 □, 32 □, 33 □, 34 □, 35 □, 36 □, 37 □, 38 □, 39 □, 40 □, 41 □, 42 □, 43 □, 44 □, 45 □, 46 □, 47 □, 48 □, 49 □, 50 □
Amaryllidaceae *Allium cepa* L. □□LI 6	Piaz	Herb	Bulb	Juice	Wound healing	Juice of plant is given 3 cups daily	29	0.16	0.034	79.31	1 ■, 2 □, 3 ■, 4 □, 5 ■, 6 ■, 7 □, 8 □, 9 ■, 10 □, 11 □, 12 □, 13 □, 14 □, 15 □, 16 □, 17 ■, 18 ●, 19□, 20 □, 21□, 22 □, 23 □, 24 ●, 25 □, 26 □, 27, 29 □, 30 □, 31 □, 32 □, 33 ■, 34 □, 35 □, 36 □, 37 □, 38 □, 39 □, 40 □, 41 □, 42 □, 43 □, 44 □, 45 □, 46 □, 47 □, 48 □, 49 ■, 50 □
Amaryllidaceae *Allium sativum* L LI 7	Lehsan	Herb	Leaf	Paste	Pimples	Paste of plant is added in different a edibles for pimples	36	0.20	0.028	91.67	1 ●, 2 □, 3 ■, 4 □, 5 □, 6 ●, 7 □, 8 □, 9 □, 10 □, 11 □, 12 □, 13 □, 14 □, 15 □, 16 □, 17 □, 18 □, 19□, 20 □, 21□, 22 □, 23 □, 24 □, 25 □, 26 □, 27 □, 28 □, 29 □, 30 □, 31 □, 32 □, 33 □, 34 □, 35 □, 36 □, 37 □, 38 □, 39 □, 40 ●, 41 □, 42 □, 43 □, 44 □, 45 □, 46 □, 47 □, 48 □, 49 ■, 50 □
Apiaceae *Anethum graveolens* L. LI 10	Soye	Herb	Whole plant	Decoction	pimples	3 cups of decoction at two time is given twice a week	20	0.11	0.050	75.00	1 □, 2 □, 3 □, 4 □, 5 □, 6 □, 7 □, 8 □, 9 □, 10 □, 11 □, 12 □, 13 □, 14 □, 15 □, 16 □, 17 □, 18 □, 19□, 20 □, 21□, 22 □, 23 □, 24 □, 25 □, 26 □, 27 □, 28 □, 29 □, 30 □, 31 □, 32 □, 33 □, 34 □, 35 □, 36 □, 37 □, 38 □, 39 □, 40 □, 41 □, 42 □, 43 □, 44 □, 45 □, 46 □, 47 □, 48 □, 49 □, 50 □
Apiaceae *Coriandrum sativum* L*.* LI 33	Dhania	Herb	Whole plant	Raw, cooked	Pimples	Whole plant as it is or add in different dishes while cooking to cure pimples	32	0.18	0.031	87.50	1 □, 2 □, 3 □, 4 □, 5 □, 6 □, 7 □, 8 □, 9 ●, 10 □, 11 □, 12 □, 13 □, 14 □, 15 □, 16 □, 17 □, 18 □, 19□, 20 □, 21□, 22 □, 23 □, 24 □, 25 □, 26 □, 27 □, 28 □, 29 □, 30 □, 31 □, 32 □, 33 □, 34 □, 35 □, 36 □, 37 □, 38 □, 39 □, 40 □, 41 □, 42 □, 43 □, 44 □, 45 □, 46 □, 47 □, 48 □, 49 □, 50 □
Apiaceae *Ferula foetida* (Bunge) Regel. LI 47		Herb	Latex	Poultice	Wound healing	Its poultice is used for wound cure	40	0.22	0.025	92.50	1 □, 2 □, 3 □, 4 □, 5 □, 6 □, 7 □, 8 □, 9 □, 10 □, 11 □, 12 □, 13 □, 14 □, 15 □, 16 □, 17 □, 18 □, 19□, 20 □, 21□, 22 □, 23 □, 24 □, 25 □, 26 □, 27 □, 28 □, 29 □, 30 □, 31 □, 32 □, 33 □, 34 □, 35 □, 36 □, 37 □, 38 □, 39 □, 40 □, 41 □, 42 □, 43 □, 44 □, 45 □, 46 □, 47 □, 48 □, 49 □, 50 □
Apiaceae *Pleurospermum brunonis* Benth. ex C.B.Clarke LI 80	Spairkai		Leaf	Powder	Skin problems	Crushed leaves are mixed in oil and applied on the skin to prevent infections.	28	0.16	0.036	85.71	1 □, 2 □, 3 □, 4 □, 5 □, 6 □, 7 □, 8 □, 9 □, 10 □, 11 □, 12 □, 13 □, 14 □, 15 □, 16 □, 17 □, 18 □, 19□, 20 ■, 21□, 22 □, 23 □, 24 □, 25 □, 26 □, 27 □, 28 □, 29 □, 30 □, 31 □, 32 □, 33 □, 34 □, 35 □, 36 □, 37 □, 38 □, 39 □, 40 □, 41 □, 42 □, 43 □, 44 □, 45 □, 46 □, 47 □, 48 □, 49 □, 50 □
Apocynaceae *Calotropis procera* (Aiton) Dryand. LI 24	Desi aak	Herb	Flower and branches	Decoction	inflammation	The decoction of flowers with honey in two ounce is given once a day	18	0.10	0.056	61.11	1 □, 2 □, 3 □, 4 □, 5 ■, 6 □, 7 □, 8 □, 9 ●, 10 □, 11 □, 12 □, 13 □, 14 □, 15 □, 16 □, 17 □, 18 □, 19□, 20 □, 21□, 22 □, 23 □, 24 □, 25 □, 26 □, 27 □, 28 □, 29 ●, 30 □, 31 □, 32 □, 33 □, 34 □, 35 □, 36 □, 37 □, 38 □, 39 □, 40 □, 41 □, 42 □, 43 □, 44 ■, 45 □, 46 □, 47 □, 48 □, 49 □, 50 □
Apocynaceae *Carissa spinarum* L. Haines LI 22	Granda	Shrub	Root, bark, Leaf	Paste	Wound healing, boil	The paste prepared from bark and root is applied on wounds for healing	25	0.14	0.080	80.00	1 □, 2 □, 3 □, 4 □, 5 □, 6 □, 7 □, 8 □, 9 □, 10 □, 11 □, 12 □, 13 □, 14 □, 15 □, 16 □, 17 □, 18 □, 19□, 20 □, 21□, 22 □, 23 □, 24 □, 25 □, 26 □, 27 □, 28 □, 29 □, 30 □, 31 □, 32 □, 33 □, 34 □, 35 □, 36 □, 37 □, 38 □, 39 □, 40 □, 41 □, 42 □, 43 □, 44 □, 45 □, 46 □, 47 □, 48 □, 49 □, 50 □
Apocynaceae *Rauvolfia serpentina* L. LI 84	Tilian	Shrub	Leaf	Extract	Skin problem	Extract or paste prepared from flower and leaf is used to cure anemia, skin diseases and blood purification	22	0.12	0.045	86.36	1 □, 2 □, 3 □, 4 □, 5 □, 6 □, 7 □, 8 □, 9 □, 10 □, 11 □, 12 □, 13 □, 14 □, 15 □, 16 □, 17 □, 18 □, 19□, 20 □, 21□, 22 □, 23 □, 24 □, 25 □, 26 □, 27 □, 28 □, 29 □, 30 □, 31 □, 32 □, 33 □, 34 □, 35 □, 36 □, 37 □, 38 □, 39 □, 40 □, 41 □, 42 □, 43 □, 44 □, 45 □, 46 □, 47 □, 48 □, 49 □, 50 □
Asteraceae *Anaphalis margaritacea* (L.) Benth. & Hook.f. LI8		Herb	Whole plant, flowers	Paste	Skin burn	Poultice made of whole plant is useful for skin burns	32	0.18	0.031	81.25	1 □, 2 □, 3 □, 4 □, 5 □, 6 □, 7 □, 8 □, 9 □, 10 □, 11 □, 12 □, 13 □, 14 □, 15 □, 16 □, 17 □, 18 □, 19□, 20 □, 21□, 22 □, 23 □, 24 □, 25 □, 26 □, 27 □, 28 □, 29 □, 30 □, 31 □, 32 □, 33 □, 34 □, 35 □, 36 □, 37 □, 38 □, 39 □, 40 □, 41 □, 42 □, 43 □, 44 □, 45 □, 46 □, 47 □, 48 □, 49 □, 50 □
Asteraceae *Artemisia vulgaris* L. LI 12	Jaokay	Herb	Leaf	Powders	boils	Dried leaves are grinded to fine powder and taken 3 spoons in the early morning.	45	0.25	0.022	80.00	1 ■, 2 □, 3 □, 4 □, 5 □, 6 □, 7 □, 8 □, 9 □, 10 ■, 11 □, 12 □, 13 □, 14 □, 15 □, 16 □, 17 □, 18 □, 19□, 20 □, 21□, 22 □, 23 □, 24 □, 25 □, 26 □, 27 □, 28 □, 29 □, 30 □, 31 □, 32 □, 33 □, 34 □, 35 □, 36 □, 37 □, 38 □, 39 □, 40 □, 41 □, 42 □, 43 □, 44 □, 45 □, 46 □, 47 □, 48 □, 49 □, 50 □
Asteraceae *Gerbera gossypina* (Royle) Beauverd LI 50	Kofe	Herb	Roots	Paste	Wound healing	Paste prepared from roots is applied to newly cut wounds to control the bleeding.	39	0.22	0.026	69.23	1 □, 2 □, 3 □, 4 □, 5 □, 6 □, 7 □, 8 □, 9 □, 10 □, 11 □, 12 □, 13 □, 14 □, 15 □, 16 □, 17 □, 18 □, 19□, 20 □, 21□, 22 □, 23 □, 24 □, 25 □, 26 □, 27 □, 28 □, 29 □, 30 □, 31 □, 32 □, 33 □, 34 □, 35 □, 36 □, 37 □, 38 □, 39 □, 40 □, 41 □, 42 □, 43 □, 44 □, 45 □, 46 □, 47 □, 48 □, 49 □, 50 □
Asteraceae *Gnaphalium affine* D.Don LI 51	Jangli dodal	Herb	Leaf	Decoction	Skin problems	A decoction made from leaves is used to cure sore throat, influenza and weeping pruritus of the skin.	12	0.07	0.083	50.00	1 □, 2 □, 3 □, 4 □, 5 □, 6 □, 7 □, 8 □, 9 □, 10 □, 11 □, 12 □, 13 □, 14 □, 15 □, 16 □, 17 □, 18 □, 19□, 20 □, 21□, 22 □, 23 □, 24 □, 25 □, 26 □, 27 □, 28 □, 29 □, 30 □, 31 □, 32 □, 33 □, 34 □, 35 □, 36 □, 37 □, 38 □, 39 □, 40 □, 41 □, 42 □, 43 □, 44 □, 45 □, 46 □, 47 □, 48 □, 49 □, 50 □
Asteraceae *Launaea nudicaulis* (L.) Hook.f. LI 60/	Herb		Leaf	Powder	Wound healing	Dried leaves are powdered and taken with water twice a day.	19	0.11	0.053	78.95	1 □, 2 □, 3 □, 4 □, 5 □, 6 □, 7 □, 8 □, 9 □, 10 □, 11 □, 12 □, 13 □, 14 □, 15 □, 16 □, 17 □, 18 □, 19□, 20 □, 21□, 22 □, 23 □, 24 □, 25 □, 26 □, 27 □, 28 □, 29 □, 30 □, 31 □, 32 □, 33 □, 34 □, 35 □, 36 □, 37 □, 38 □, 39 □, 40 □, 41 □, 42 □, 43 □, 44 □, 45 □, 46 □, 47 □, 48 □, 49 □, 50 □
Asteraceae *Saussurea lappa* (Decne.) Sch.Bip. LI 93		Herb	Roots	Extract	Skin problem	Tonic, carminative, used in cholera and in chronic skin problems	39	0.22	0.026	76.92	1 □, 2 □, 3 □, 4 □, 5 □, 6 □, 7 □, 8 □, 9 □, 10 □, 11 □, 12 □, 13 □, 14 □, 15 □, 16 □, 17 □, 18 □, 19□, 20 □, 21□, 22 □, 23 □, 24 □, 25 □, 26 □, 27 □, 28 □, 29 □, 30 □, 31 □, 32 □, 33 □, 34 □, 35 □, 36 □, 37 □, 38 □, 39 □, 40 □, 41 □, 42 □, 43 □, 44 □, 45 □, 46 □, 47 □, 48 □, 49 □, 50 □
Asteraceae *Senecio chrysanthemoides* DC LI 94		Herb	Leaf	Oil	Skin problem	Oil is used for treatment	36	0.20	0.056	80.56	1 □, 2 □, 3 □, 4 □, 5 □, 6 □, 7 □, 8 □, 9 □, 10 □, 11 □, 12 □, 13 □, 14 □, 15 □, 16 □, 17 □, 18 □, 19□, 20 □, 21□, 22 □, 23 □, 24 □, 25 □, 26 □, 27 □, 28 □, 29 □, 30 □, 31 □, 32 □, 33 □, 34 □, 35 □, 36 □, 37 □, 38 □, 39 □, 40 □, 41 □, 42 □, 43 □, 44 □, 45 □, 46 □, 47 □, 48 □, 49 □, 50 □
Asteraceae *Sonchus asper* (L.) Hill LI 96		Herb	Flower, Leaf	Powder	Skin problem	Dried flowers and leaves are powdered and taken for the treatment of rheumatism.	26	0.14	0.038	100.00	1 □, 2 □, 3 □, 4 □, 5 □, 6 □, 7 □, 8 □, 9 □, 10 □, 11 □, 12 □, 13 □, 14 □, 15 □, 16 □, 17 □, 18 □, 19□, 20 □, 21□, 22 □, 23 □, 24 □, 25 □, 26 □, 27 □, 28 □, 29 □, 30 □, 31 □, 32 □, 33 □, 34 □, 35 □, 36 □, 37 □, 38 □, 39 □, 40 □, 41 □, 42 □, 43 □, 44 □, 45 □, 46 □, 47 □, 48 □, 49 □, 50 □
Asteraceae *Taraxacum officinale* aggr. F.H. Wigg. LI 99	Haand	Herb	Flowers, Leaf, roots	Tea	Pimples	The tea prepared from flowers is used internally to cure pimples and is used cosmetically to clear the skin	35	0.19	0.029	94.29	1 □, 2 □, 3 □, 4 □, 5 □, 6 □, 7 □, 8 □, 9 □, 10 □, 11 □, 12 □, 13 □, 14 □, 15 □, 16 □, 17 □, 18 □, 19□, 20 □, 21□, 22 □, 23 □, 24 □, 25 □, 26 □, 27 □, 28 □, 29 □, 30 □, 31 □, 32 □, 33 □, 34 □, 35 □, 36 □, 37 □, 38 □, 39 □, 40 □, 41 □, 42 □, 43 □, 44 □, 45 □, 46 □, 47 □, 48 □, 49 □, 50 □
Asteraceae *Tussilago farfara* L. LI 103	Bann Hulla		Flowers	Poultice	Skin problems	A poultice made from flowers is used for the treatment of a range of skin disorders including ulcers, sores, and Inflammations.	27	0.15	0.037	77.78	1 □, 2 □, 3 □, 4 □, 5 □, 6 □, 7 □, 8 □, 9 □, 10 □, 11 □, 12 □, 13 □, 14 □, 15 □, 16 □, 17 □, 18 □, 19□, 20 □, 21□, 22 □, 23 □, 24 □, 25 □, 26 □, 27 □, 28 □, 29 □, 30 □, 31 □, 32 □, 33 □, 34 □, 35 □, 36 □, 37 □, 38 □, 39 □, 40 □, 41 □, 42 □, 43 □, 44 □, 45 □, 46 □, 47 □, 48 □, 49 □, 50 □
Balsaminaceae *Impatien edgeworthii* Hook. f LI 54	Buntil	Herb	Whole plant	Paste	Skin burn	The plant paste is used externally for burns	33	0.18	0.030	81.82	1 □, 2 □, 3 □, 4 □, 5 □, 6 □, 7 □, 8 □, 9 □, 10 □, 11 □, 12 □, 13 □, 14 □, 15 □, 16 □, 17 □, 18 □, 19□, 20 □, 21□, 22 □, 23 □, 24 □, 25 □, 26 □, 27 □, 28 □, 29 □, 30 □, 31 □, 32 □, 33 □, 34 □, 35 □, 36 □, 37 □, 38 □, 39 □, 40 □, 41 □, 42 □, 43 □, 44 □, 45 □, 46 □, 47 □, 48 □, 49 □, 50 □
Berberidaceae *Berberis lycium* Royle LI 15	Sumblu/ komal	Shrub	Leaf, root, flowers	Paste	Wound healing	The paste prepared from leaves and roots is externally applied on wounds.	21	0.12	0.048	80.95	1 □, 2 □, 3 □, 4 □, 5 ■, 6 □, 7 □, 8 □, 9 □, 10 □, 11 □, 12 □, 13 □, 14 □, 15 □, 16 □, 17 □, 18 □, 19□, 20 ●, 21□, 22 □, 23 □, 24 □, 25 □, 26 □, 27 □, 28 □, 29 □, 30 □, 31 □, 32 □, 33 □, 34 □, 35 □, 36 □, 37 □, 38 □, 39 □, 40 ■, 41 □, 42 □, 43 □, 44 □, 45 □, 46 □, 47 □, 48 □, 49 □, 50 □
Boraginaceae *Hackelia americana* (A.Gray) Fernald LI 52	Neelaan	Herb	Flowers		Wounds	The flowers are good expectorant, used for wound healing and treating tumors. Flowers are used to cure coughs, sores, and swellings.	28	0.16	0.036	78.57	1 □, 2 □, 3 □, 4 □, 5 □, 6 □, 7 □, 8 □, 9 □, 10 □, 11 □, 12 □, 13 □, 14 □, 15 □, 16 □, 17 □, 18 □, 19□, 20 □, 21□, 22 □, 23 □, 24 □, 25 □, 26 □, 27 □, 28 □, 29 □, 30 □, 31 □, 32 □, 33 □, 34 □, 35 □, 36 □, 37 □, 38 □, 39 □, 40 □, 41 □, 42 □, 43 □, 44 □, 45 □, 46 □, 47 □, 48 □, 49 □, 50 □
Boraginaceae *Onosma hispida* Wall. ex G. LI 71	Lal jari	Tree	Leaf, Flower, Roots	Poultice	Skin burn	Leaf poultice are applied on the Burnt wounds with ghee/ oil.	33	0.18	0.030	72.73	1 □, 2 □, 3 □, 4 □, 5 □, 6 □, 7 □, 8 □, 9 □, 10 □, 11 □, 12 □, 13 □, 14 □, 15 □, 16 □, 17 □, 18 □, 19□, 20 □, 21□, 22 □, 23 □, 24 □, 25 □, 26 □, 27 □, 28 □, 29 □, 30 □, 31 □, 32 □, 33 □, 34 □, 35 □, 36 □, 37 □, 38 □, 39 □, 40 □, 41 □, 42 □, 43 □, 44 □, 45 □, 46 □, 47 □, 48 □, 49 □, 50 □
Brassicaceae *Brassica juncea* (L.) Czern. LI 20	Sharsham	Herb	Leaf	Cooked	Wound healing	Leaf are cooked and used for wound healing	21	0.12	0.048	66.67	1 □, 2 □, 3 □, 4 □, 5 □, 6 □, 7 □, 8 □, 9 □, 10 □, 11 □, 12 □, 13 □, 14 □, 15 □, 16 □, 17 □, 18 □, 19□, 20 □, 21□, 22 □, 23 □, 24 □, 25 □, 26 □, 27 □, 28 □, 29 □, 30 □, 31 □, 32 □, 33 □, 34 □, 35 □, 36 □, 37 □, 38 □, 39 □, 40 □, 41 □, 42 □, 43 □, 44 □, 45 □, 46 □, 47 □, 48 □, 49 □, 50 □
Buxaceae *Buxus papillosa* C.K. Schneid. LI 21	Angaroo	Shrub	Leaf	Oil	Skin problems	Oil of Leaf are applied on skin	29	0.16	0.034	79.31	1 □, 2 □, 3 □, 4 □, 5 □, 6 □, 7 □, 8 ■, 9 □, 10 □, 11 □, 12 □, 13 □, 14 □, 15 □, 16 □, 17 □, 18 □, 19□, 20 □, 21□, 22 □, 23 □, 24 □, 25 □, 26 □, 27 □, 28 □, 29 □, 30 □, 31 □, 32 ■, 33 ■, 34 □, 35 □, 36 □, 37 □, 38 □, 39 □, 40 □, 41 □, 42 □, 43 □, 44 □, 45 □, 46 □, 47 □, 48 □, 49 □, 50 □
Cannabaceae *Cannabis sativa* L LI 26	Bhang	Shrub	Flower, fruit, Leaf	Juice, powder	Dandruff, wounds healing	The fresh juice of Leaf and flowers are used for removing dandruff Fr.om the head. Powder of the Leaf and fruits are beneficial for dressing fresh wounds	17	0.09	0.118	94.12	1 □, 2 □, 3 □, 4 □, 5 □, 6 □, 7 □, 8 □, 9 ■, 10 □, 11 □, 12 □, 13 □, 14 □, 15 □, 16 □, 17 □, 18 □, 19□, 20 □, 21□, 22 □, 23 □, 24 □, 25 □, 26 □, 27 □, 28 □, 29 □, 30 □, 31 □, 32 □, 33 □, 34 □, 35 □, 36 □, 37 □, 38 □, 39 □, 40 □, 41 □, 42 □, 43 □, 44 □, 45 ●, 46 □, 47 □, 48 □, 49 □, 50 □
Capparaceae *Capparis decidua* (Forssk.) Edgew. LI 27	Keera	Tree	Seeds	Decoction	Wound healing	Decoction prepared from seeds is taken 3 cups daily to cure wounds.	24	0.13	0.042	91.67	1 □, 2 □, 3 □, 4 □, 5 □, 6 □, 7 □, 8 □, 9 □, 10 □, 11 □, 12 □, 13 □, 14 □, 15 □, 16 □, 17 □, 18 □, 19□, 20 □, 21□, 22 □, 23 □, 24 □, 25 □, 26 □, 27 □, 28 □, 29 □, 30 □, 31 □, 32 □, 33 □, 34 □, 35 □, 36 □, 37 □, 38 □, 39 □, 40 □, 41 □, 42 □, 43 □, 44 □, 45 □, 46 □, 47 □, 48 □, 49 □, 50 □
Caprifoliaceae *Valeriana jatamansi* Jones ex Roxb. LI 105	Murma		Roots	Juice	Pimples	The root juice is used to cure hysteria, pimples, rheumatism, nausea and cholera	22	0.12	0.045	86.36	1 □, 2 □, 3 □, 4 □, 5 □, 6 □, 7 □, 8 □, 9 □, 10 □, 11 □, 12 □, 13 □, 14 □, 15 □, 16 □, 17 □, 18 □, 19□, 20 □, 21□, 22 □, 23 □, 24 □, 25 □, 26 □, 27 □, 28 □, 29 □, 30 □, 31 □, 32 □, 33 □, 34 □, 35 □, 36 □, 37 □, 38 □, 39 □, 40 □, 41 □, 42 □, 43 □, 44 □, 45 □, 46 □, 47 □, 48 □, 49 □, 50 □
Caryophyllaceae *Cerastium fontanum subsp. vulgare* (Hartm.) Greuter & Burdet, LI 29			Bark	Powder	Skin problem	Powdered bark along with milk is taken orally at morning to treat skin problems.	38	0.21	0.026	89.47	1 □, 2 □, 3 □, 4 □, 5 □, 6 □, 7 □, 8 □, 9 □, 10 □, 11 □, 12 □, 13 □, 14 □, 15 □, 16 □, 17 □, 18 □, 19□, 20 □, 21□, 22 □, 23 □, 24 □, 25 □, 26 □, 27 □, 28 □, 29 □, 30 □, 31 □, 32 □, 33 □, 34 □, 35 □, 36 □, 37 □, 38 □, 39 □, 40 □, 41 □, 42 □, 43 □, 44 □, 45 □, 46 □, 47 □, 48 □, 49 □, 50 □
Commelinaceae *Commelina benghalensis* L LI 32	Chora	Herb	Leaf, Fruit	Raw	Wound infection	Whole fruit is used to treat wounds	33	0.18	0.030	84.85	1 □, 2 □, 3 □, 4 □, 5 □, 6 □, 7 □, 8 □, 9 ■, 10 □, 11 □, 12 □, 13 □, 14 □, 15 □, 16 □, 17 □, 18 □, 19□, 20 □, 21□, 22 □, 23 □, 24 □, 25 □, 26 □, 27 □, 28 □, 29 □, 30 □, 31 □, 32 □, 33 □, 34 □, 35 □, 36 □, 37 □, 38 □, 39 □, 40 □, 41 □, 42 □, 43 □, 44 ●, 45 □, 46 □, 47 □, 48 □, 49 □, 50 □
Convolvulaceae *Cuscta reflexa* Roxb. LI 35	Neeltharee	Tree	Roots	Decoction	Skin problems	Crushed roots are boiled in water and some sugar is added.	28	0.16	0.036	92.86	1 □, 2 □, 3 □, 4 □, 5 □, 6 □, 7 □, 8 □, 9 □, 10 □, 11 □, 12 □, 13 □, 14 □, 15 □, 16 □, 17 □, 18 □, 19□, 20 □, 21□, 22 □, 23 □, 24 □, 25 □, 26 □, 27 □, 28 □, 29 □, 30 □, 31 □, 32 □, 33 □, 34 □, 35 □, 36 □, 37 □, 38 □, 39 □, 40 □, 41 □, 42 □, 43 □, 44 □, 45 □, 46 □, 47 □, 48 □, 49 □, 50 □
Cucurbitaceae *Cucumis melo* L. LI 36	Tori	Herb	Fruit	Infusion	Skin burn	Infusion of fruits used to cure skin burns	26	0.14	0.038	92.31	1 □, 2 □, 3 □, 4 □, 5 □, 6 □, 7 □, 8 □, 9 □, 10 ■, 11 □, 12 □, 13 □, 14 □, 15 □, 16 □, 17 □, 18 □, 19□, 20 □, 21□, 22 □, 23 □, 24 □, 25 □, 26 □, 27 □, 28 □, 29 □, 30 □, 31 □, 32 □, 33 □, 34 □, 35 □, 36 □, 37 □, 38 □, 39 □, 40 □, 41 □, 42 □, 43 □, 44 □, 45 □, 46 □, 47 □, 48 □, 49 □, 50 □
Cucurbitaceae *Lagenaria siceraria* (Molina) Standl. LI 59	Gya Kadoo	Herb	Leaf, fruit	Raw	Wound healing, skin burn	Eaten daily as tonic	28	0.16	0.071	57.14	1 □, 2 □, 3 □, 4 □, 5 □, 6 □, 7 □, 8 □, 9 □, 10 □, 11 □, 12 □, 13 □, 14 □, 15 □, 16 □, 17 □, 18 □, 19□, 20 □, 21□, 22 □, 23 □, 24 □, 25 □, 26 □, 27 □, 28 □, 29 □, 30 □, 31 □, 32 □, 33 □, 34 □, 35 □, 36 □, 37 □, 38 □, 39 □, 40 □, 41 □, 42 □, 43 □, 44 □, 45 □, 46 □, 47 □, 48 □, 49 □, 50 □
Cucurbitaceae *Momordica charantia* L. LI 67	Kareela	Herb	Flowers, roots	Paste	Wound healing	Paste of herb is applied for wound healing	19	0.11	0.053	94.74	1 □, 2 □, 3 □, 4 □, 5 □, 6 □, 7 □, 8 □, 9 □, 10 □, 11 □, 12 □, 13 □, 14 □, 15 □, 16 □, 17 ■, 18 □, 19□, 20 □, 21□, 22 □, 23 □, 24 □, 25 □, 26 □, 27 □, 28 □, 29 ■, 30 □, 31 □, 32 □, 33 □, 34 □, 35 □, 36 □, 37 □, 38 □, 39 □, 40 □, 41 □, 42 □, 43 □, 44 □, 45 □, 46 □, 47 □, 48 □, 49 □, 50 □
Cupressaceae *Juniperus communis* L*.* LI 56	Gojar	Tree	Berries	Decoction	Skin problem	An ointment of berries are used in skin problem	25	0.14	0.040	76.00	1 □, 2 □, 3 □, 4 □, 5 □, 6 □, 7 □, 8 □, 9 □, 10 □, 11 □, 12 □, 13 □, 14 □, 15 □, 16 □, 17 □, 18 □, 19□, 20 □, 21□, 22 □, 23 □, 24 □, 25 □, 26 □, 27 □, 28 □, 29 □, 30 □, 31 □, 32 □, 33 □, 34 □, 35 □, 36 □, 37 □, 38 □, 39 □, 40 □, 41 □, 42 □, 43 □, 44 □, 45 □, 46 □, 47 □, 48 □, 49 □, 50 □
Cupressaceae *Juniperus excelsa* M. Bieb. LI 57	Pencil Cedar	Tree	Bark	Powder	Skin Problem	Powder of the bark is used in certain skin infection areas	11	0.06	0.091	72.73	1 □, 2 □, 3 □, 4 □, 5 □, 6 □, 7 □, 8 □, 9 □, 10 □, 11 □, 12 □, 13 □, 14 □, 15 □, 16 □, 17 □, 18 □, 19□, 20 □, 21□, 22 □, 23 □, 24 □, 25 □, 26 □, 27 □, 28 □, 29 □, 30 □, 31 □, 32 □, 33 □, 34 □, 35 □, 36 □, 37 □, 38 □, 39 □, 40 □, 41 □, 42 □, 43 □, 44 □, 45 □, 46 □, 47 □, 48 □, 49 □, 50 □
Cyperaceae *Cyperus difformis* L LI 38	Motkopragha	Herb	Whole plant	Paste	Skin problems	Paste prepared from whole plants is applied externally to cure skin infections.	14	0.08	0.071	71.43	1 □, 2 □, 3 □, 4 □, 5 □, 6 □, 7 □, 8 □, 9 □, 10 □, 11 □, 12 □, 13 □, 14 □, 15 □, 16 □, 17 □, 18 □, 19□, 20 □, 21□, 22 □, 23 □, 24 □, 25 □, 26 □, 27 □, 28 □, 29 □, 30 □, 31 □, 32 □, 33 □, 34 □, 35 □, 36 □, 37 □, 38 □, 39 □, 40 □, 41 □, 42 □, 43 □, 44 □, 45 □, 46 □, 47 □, 48 □, 49 □, 50 □
Elaeagnacea*e Hippophae rhamnoides* L. LI 53		Tree	Fruit, seeds	Decoction	Skin problems	A decoction of the fruits are used for skin problems	37	0.21	0.027	83.78	1 □, 2 □, 3 □, 4 □, 5 □, 6 □, 7 □, 8 □, 9 □, 10 □, 11 □, 12 □, 13 □, 14 □, 15 □, 16 □, 17 □, 18 □, 19□, 20 □, 21□, 22 □, 23 □, 24 □, 25 □, 26 □, 27 □, 28 ■, 29 □, 30 □, 31 □, 32 □, 33 ●, 34 □, 35 □, 36 □, 37 □, 38 □, 39 □, 40 □, 41 □, 42 □, 43 □, 44 □, 45 □, 46 □, 47 □, 48 □, 49 □, 50 □
Equisetaceae *Equisetum arvense* L. LI 43	Chew Shina	Herb	Whole Plant	Powder	Skin problems, allergy	Plant material are mixed with different herbs and used on skin troubles and allergy	36	0.20	0.056	86.11	1 □, 2 □, 3 □, 4 □, 5 □, 6 □, 7 □, 8 □, 9 □, 10 □, 11 □, 12 □, 13 □, 14 □, 15 □, 16 □, 17 □, 18 □, 19□, 20 □, 21□, 22 □, 23 □, 24 □, 25 □, 26 □, 27 □, 28 □, 29 □, 30 □, 31 □, 32 □, 33 □, 34 □, 35 □, 36 □, 37 □, 38 □, 39 □, 40 □, 41 □, 42 □, 43 □, 44 □, 45 □, 46 □, 47 □, 48 □, 49 ●, 50 □
Euphorbiaceae *Euphorbia helioscopia* L. LI 44	Cat milk	Herb	Leaf	powder	Wound healing	Dried leaves are mixed in water and taken orally for 4–5 days.	22	0.12	0.045	81.82	1 □, 2 □, 3 □, 4 □, 5 ■, 6 □, 7 □, 8 □, 9 □, 10 □, 11 □, 12 □, 13 □, 14 □, 15 □, 16 □, 17 □, 18 □, 19□, 20 □, 21□, 22 □, 23 □, 24 □, 25 □, 26 □, 27 □, 28 □, 29 □, 30 □, 31 □, 32 □, 33 □, 34 □, 35 □, 36 □, 37 □, 38 □, 39 □, 40 □, 41 □, 42 □, 43 □, 44 □, 45 □, 46 □, 47 □, 48 □, 49 □, 50 □
Fabaceae *Butea monosperma* (Lam.) Kuntze LI 14	Chichra	Tree	Root	Decoction	Skin problem	Root decoction is used in skin diseases	36	0.20	0.028	94.44	1 □, 2 □, 3 □, 4 □, 5 □, 6 □, 7 □, 8 □, 9 □, 10 □, 11 □, 12 □, 13 □, 14 □, 15 □, 16 □, 17 □, 18 □, 19□, 20 □, 21□, 22 □, 23 □, 24 □, 25 □, 26 □, 27 □, 28 □, 29 □, 30 □, 31 □, 32 □, 33 □, 34 ●, 35 □, 36 □, 37 □, 38 □, 39 □, 40 □, 41 □, 42 □, 43 □, 44 ●, 45 □, 46 □, 47 □, 48 □, 49 □, 50 □
Fabaceae *Delbergia sissoo* L. LI 41	Shesham	Tree	Leaf	Decoction, infusion	Skin problem, abscesses	Leaves are dried, mixed with water and taken orally for 4–5 days.	40	0.22	0.050	95.00	1 □, 2 □, 3 □, 4 □, 5 □, 6 □, 7 □, 8 □, 9 □, 10 □, 11 □, 12 □, 13 □, 14 □, 15 □, 16 □, 17 □, 18 □, 19□, 20 □, 21□, 22 □, 23 □, 24 □, 25 □, 26 □, 27 □, 28 □, 29 □, 30 □, 31 □, 32 □, 33 □, 34 □, 35 □, 36 □, 37 □, 38 □, 39 □, 40 □, 41 □, 42 □, 43 □, 44 □, 45 □, 46 □, 47 □, 48 □, 49 □, 50 □
Fabaceae *Pisum sativum* L. LI 77	Matar	Herb	Seed	Extract	Skin burn	Fresh seeds are milled then extract drops being used.	21	0.12	0.143	80.95	1 □, 2 □, 3 □, 4 □, 5 □, 6 □, 7 □, 8 □, 9 □, 10 □, 11 □, 12 □, 13 □, 14 □, 15 □, 16 □, 17 □, 18 □, 19□, 20 □, 21□, 22 □, 23 □, 24 □, 25 □, 26 □, 27 □, 28 □, 29 □, 30 □, 31 □, 32 □, 33 □, 34 □, 35 □, 36 □, 37 □, 38 □, 39 □, 40 □, 41 □, 42 □, 43 □, 44 □, 45 □, 46 □, 47 □, 48 □, 49 □, 50 □
Fabaceae *Trigonella foenum-graecum* L LI 102	Jangli	Herb	Leaf, flowers	Decoction	Wound healing	Leaf and flowers are boiled in water used for cure wounds	39	0.22	0.026	82.05	1 □, 2 □, 3 □, 4 □, 5 □, 6 □, 7 □, 8 □, 9 □, 10 □, 11 □, 12 □, 13 □, 14 □, 15 ■, 16 □, 17 □, 18 □, 19□, 20 □, 21□, 22 □, 23 □, 24 □, 25 □, 26 □, 27 □, 28 ■, 29 □, 30 □, 31 □, 32 □, 33 ●, 34 □, 35 □, 36 □, 37 □, 38 □, 39 □, 40 □, 41 □, 42 □, 43 □, 44 □, 45 □, 46 □, 47 □, 48 □, 49 □, 50 □
Gentianaceae *Swertia abyssinica* Hochst. LI 97	Chratia	Shrub	Flower, Leaf	Paste	Skin problems	Plant is crushed into paste and applied on skin.	17	0.09	0.059	94.12	1 □, 2 □, 3 □, 4 □, 5 □, 6 □, 7 □, 8 □, 9 □, 10 □, 11 □, 12 □, 13 □, 14 □, 15 □, 16 □, 17 □, 18 □, 19□, 20 □, 21□, 22 □, 23 □, 24 □, 25 □, 26 □, 27 □, 28 □, 29 □, 30 □, 31 □, 32 □, 33 □, 34 □, 35 □, 36 □, 37 □, 38 □, 39 □, 40 □, 41 □, 42 □, 43 □, 44 □, 45 □, 46 □, 47 □, 48 □, 49 □, 50 □
Lamiaceae *Ajuga integrifolia* Buch-Ham-ex D. Don LI 5	Bootei	Herb	Leaf	Powder	Boils	One table spoon of powdered leaves is taken for boils treatment on daily basis.	22	0.12	0.045	81.82	1 □, 2 □, 3 □, 4 □, 5 □, 6 □, 7 □, 8 □, 9 □, 10 □, 11 □, 12 □, 13 □, 14 □, 15 □, 16 □, 17 □, 18 □, 19□, 20 □, 21□, 22 □, 23 □, 24 □, 25 □, 26 □, 27 □, 28 □, 29 □, 30 □, 31 □, 32 □, 33 □, 34 □, 35 □, 36 □, 37 □, 38 □, 39 □, 40 □, 41 □, 42 □, 43 □, 44 □, 45 □, 46 □, 47 □, 48 □, 49 □, 50 □
Lamiaceae *Isodon rugosus* (Wall. ex Benth.) LI 55	Sperkay	Shrub	Leaf	Powder	Wound healing	Powdered leaves are taken 3 times a day after each meal.	20	0.11	0.050	90.00	1 □, 2 □, 3 □, 4 □, 5 □, 6 □, 7 □, 8 □, 9 □, 10 □, 11 □, 12 □, 13 □, 14 □, 15 □, 16 □, 17 □, 18 □, 19□, 20 □, 21□, 22 □, 23 □, 24 □, 25 □, 26 □, 27 □, 28 □, 29 □, 30 □, 31 □, 32 □, 33 □, 34 □, 35 □, 36 □, 37 □, 38 □, 39 □, 40 □, 41 □, 42 □, 43 □, 44 □, 45 □, 46 □, 47 □, 48 □, 49 □, 50 □
Lamiaceae *Micromeria biflora* (Buch.-Ham. ex D.Don) Benth LI 66	Narayshamakay	Herb	Flowers, Leaf, roots	Paste	Wound healing	Root Leaf and flower paste is used for poultice making to treat wounds.	15	0.08	0.067	60.00	1 □, 2 □, 3 □, 4 □, 5 □, 6 □, 7 □, 8 □, 9 □, 10 □, 11 □, 12 □, 13 □, 14 □, 15 □, 16 □, 17 □, 18 □, 19□, 20 □, 21□, 22 □, 23 □, 24 □, 25 □, 26 □, 27 □, 28 □, 29 □, 30 □, 31 □, 32 □, 33 □, 34 □, 35 □, 36 □, 37 □, 38 □, 39 □, 40 □, 41 □, 42 □, 43 □, 44 □, 45 □, 46 □, 47 □, 48 □, 49 □, 50 □
Lamiaceae *Nepeta hindostana* (B.Heyne ex Roth) Haines. LI 68	Indian catnip	Herb	Leaf	Extract	Skin problems	The leaf extract is prepared and one small teaspoon is taken twice a day.	21	0.12	0.048	80.95	1 □, 2 □, 3 □, 4 □, 5 □, 6 □, 7 □, 8 □, 9 □, 10 □, 11 □, 12 □, 13 □, 14 □, 15 □, 16 □, 17 □, 18 □, 19□, 20 □, 21□, 22 □, 23 □, 24 □, 25 □, 26 □, 27 □, 28 □, 29 □, 30 □, 31 □, 32 □, 33 □, 34 □, 35 □, 36 □, 37 □, 38 □, 39 □, 40 □, 41 □, 42 □, 43 □, 44 □, 45 □, 46 □, 47 □, 48 □, 49 □, 50 □
Lamiaceae *Rydingia limbata* (Benth.) Scheen & V.A. Albert LI 90	Ghawareja	Shrub	Leaf	Extract	Skin problem	Leaves extract is taken orally to cure mouth ulcers and skin disorders.	23	0.13	0.043	100.00	1 □, 2 □, 3 □, 4 □, 5 □, 6 □, 7 □, 8 □, 9 □, 10 □, 11 □, 12 □, 13 □, 14 □, 15 □, 16 □, 17 □, 18 □, 19□, 20 □, 21□, 22 □, 23 □, 24 □, 25 □, 26 □, 27 □, 28 □, 29 □, 30 □, 31 □, 32 □, 33 □, 34 □, 35 □, 36 □, 37 □, 38 □, 39 □, 40 □, 41 □, 42 □, 43 □, 44 □, 45 □, 46 □, 47 □, 48 □, 49 □, 50 □
Lamiaceae *Salvia moorcroftiana* wall. ex Benth LI 92	Khaar dug, Zarshal,	Herb	Leaf	Poultice	Wound healing, skin itching	Poultice of the Leaf are used for external skin itching	17	0.09	0.059	64.71	1 □, 2 □, 3 □, 4 □, 5 ■, 6 □, 7 □, 8 □, 9 □, 10 □, 11 □, 12 □, 13 □, 14 □, 15 □, 16 □, 17 □, 18 □, 19□, 20 □, 21□, 22 □, 23 □, 24 □, 25 □, 26 □, 27 □, 28 □, 29 □, 30 □, 31 □, 32 □, 33 □, 34 □, 35 □, 36 □, 37 □, 38 □, 39 □, 40 □, 41 □, 42 □, 43 □, 44 □, 45 □, 46 □, 47 □, 48 □, 49 □, 50 □
Lamiaceae *Teucrium stocksianum* Boiss. LI 101	Kwandi Bootay	Herb	Leaf	Decoction	Wound healing	Decoction of Leaf is employed in wound healing.	25	0.14	0.040	88.00	1 □, 2 □, 3 □, 4 □, 5 □, 6 □, 7 □, 8 □, 9 □, 10 □, 11 □, 12 □, 13 □, 14 □, 15 □, 16 □, 17 □, 18 □, 19□, 20 □, 21□, 22 □, 23 □, 24 □, 25 □, 26 □, 27 □, 28 □, 29 □, 30 □, 31 □, 32 □, 33 □, 34 □, 35 □, 36 □, 37 □, 38 □, 39 □, 40 □, 41 □, 42 □, 43 □, 44 □, 45 □, 46 □, 47 □, 48 □, 49 □, 50 □
Loranthaceae *Loranthus pulverulentus* Wall LI 62	Parwikh	Shrub	Leaf	Powder	Wound healing	Leaf powder is used for wound healing.	32	0.18	0.031	71.88	1 □, 2 □, 3 □, 4 □, 5 □, 6 □, 7 □, 8 □, 9 □, 10 □, 11 □, 12 □, 13 □, 14 □, 15 □, 16 □, 17 □, 18 □, 19□, 20 □, 21□, 22 □, 23 □, 24 □, 25 □, 26 □, 27 □, 28 □, 29 □, 30 □, 31 □, 32 □, 33 □, 34 □, 35 □, 36 □, 37 □, 38 □, 39 □, 40 □, 41 □, 42 □, 43 □, 44 □, 45 □, 46 □, 47 □, 48 □, 49 □, 50 □
Lythraceae *Lawsonia inermis* L. LI 61	Mhendi	Shrub	Leaves	Infusion	Skin burn, boils	Crushed leaves are dissolved in water and infusion made is taken for 4–5 days	39	0.22	0.051	61.54	1 ■, 2 ●, 3 ■, 4 □, 5 □, 6 □, 7 □, 8 □, 9 ■, 10 ●, 11 □, 12 □, 13 □, 14 □, 15 ■, 16 □, 17 □, 18 ■, 19□, 20 □, 21□, 22 □, 23 □, 24 □, 25 □, 26 □, 27 □, 28 □, 29 ■, 30 □, 31 □, 32 □, 33 □, 34 □, 35 □, 36 □, 37 □, 38 □, 39 □, 40 □, 41 □, 42 □, 43 □, 44 □, 45 □, 46 □, 47 □, 48 □, 49 ●, 50 □
Malvaceae *Abelmoschus esculentus* (L.) Moench LI 1	Bhindi	Herb	Seeds	Tea	pimples	Seeds are boil in water and make tea which is used in pimples cure	29	0.16	0.034	72.41	1 □, 2 □, 3 □, 4 □, 5 □, 6 □, 7 □, 8 □, 9 □, 10 □, 11 □, 12 □, 13 □, 14 □, 15 □, 16 □, 17 □, 18 □, 19□, 20 □, 21□, 22 □, 23 □, 24 □, 25 □, 26 □, 27 □, 28 □, 29 □, 30 □, 31 □, 32 □, 33 □, 34 □, 35 □, 36 □, 37 □, 38 □, 39 □, 40 □, 41 □, 42 □, 43 □, 44 □, 45 □, 46 □, 47 □, 48 □, 49 □, 50 □
Meliaceae *Melia azadarach* L. LI 65	Draik	Tree	Leaf	Powder	Pimples, Inflammation	Three teaspoons of grinded leaves are mixed in three cups of hot water and used twice a day.	27	0.15	0.074	74.07	1 □, 2 □, 3 □, 4 □, 5 ■, 6 □, 7 □, 8 □, 9 □, 10 □, 11 □, 12 □, 13 □, 14 □, 15 □, 16 □, 17 □, 18 □, 19□, 20 □, 21□, 22 □, 23 □, 24 □, 25 □, 26 □, 27 □, 28 □, 29 □, 30 □, 31 □, 32 □, 33 □, 34 □, 35 □, 36 □, 37 □, 38 □, 39 □, 40 □, 41 □, 42 □, 43 □, 44 □, 45 □, 46 □, 47 □, 48 □, 49 □, 50 □
Myrsinaceae *Myrsine africana* L. LI 63/	Gugal	Shrub	Leaf		Skin problems	Leaves are used to cure cough, cold, flue and skin disorders.	35	0.19	0.029	91.43	1 □, 2 □, 3 □, 4 □, 5 ■, 6 □, 7 □, 8 □, 9 □, 10 □, 11 □, 12 □, 13 □, 14 □, 15 □, 16 □, 17 □, 18 □, 19□, 20 □, 21□, 22 □, 23 □, 24 □, 25 □, 26 □, 27 □, 28 □, 29 □, 30 □, 31 □, 32 □, 33 □, 34 □, 35 □, 36 □, 37 □, 38 □, 39 □, 40 □, 41 □, 42 □, 43 □, 44 □, 45 □, 46 □, 47 □, 48 □, 49 □, 50 □
Nitrariaceae *Peganum harmala* L. LI 72	Isman	Herb	Leaf	Extract	Skin problem	The aqueous extract of leaves is used thrice a day to treat skin problems.	35	0.19	0.029	65.71	1 □, 2 □, 3 □, 4 □, 5 □, 6 □, 7 □, 8 □, 9 □, 10 □, 11 □, 12 □, 13 □, 14 □, 15 □, 16 □, 17 □, 18 □, 19□, 20 □, 21□, 22 ■, 23 □, 24 □, 25 □, 26 □, 27 □, 28 □, 29 □, 30 □, 31 □, 32 □, 33 ■, 34 □, 35 □, 36 □, 37 □, 38 □, 39 □, 40 □, 41 □, 42 □, 43 □, 44 □, 45 □, 46 □, 47 □, 48 □, 49 □, 50 □
Nyctaginaceae *Boerrehavia diffusa* L. LI 19/	Snnati	Herb	Leaf	Infusion	abscesses	Leaves are crushed and added in water, used to cure skin abscission.	27	0.15	0.037	81.48	1 □, 2 □, 3 □, 4 □, 5 □, 6 □, 7 □, 8 □, 9 □, 10 □, 11 □, 12 □, 13 □, 14 □, 15 □, 16 □, 17 □, 18 □, 19□, 20 □, 21□, 22 □, 23 □, 24 □, 25 □, 26 □, 27 □, 28 □, 29 □, 30 □, 31 □, 32 □, 33 □, 34 □, 35 □, 36 □, 37 □, 38 □, 39 □, 40 □, 41 □, 42 □, 43 □, 44 □, 45 □, 46 □, 47 □, 48 □, 49 □, 50 □
Oleaceae *Olea europaea subsp. cuspidata* (Wall. & G.Don) Cif LI 70	Ghawareja	Shrub	Leaf,seeds	Tea	Skin problems	Leaves are boiled and the tea is taken orally to cure mouth ulcers and skin disorders.	31	0.17	0.032	80.65	1 ■, 2 □, 3 □, 4 □, 5 □, 6 □, 7 ■, 8 ●, 9 □, 10 □, 11 □, 12 □, 13 □, 14 □, 15 □, 16 □, 17 □, 18 □, 19□, 20 □, 21□, 22 □, 23 □, 24 □, 25 □, 26 □, 27 □, 28 ●, 29 □, 30 □, 31 □, 32 ●, 33 □, 34 □, 35 □, 36 □, 37 □, 38 □, 39 □, 40 □, 41 □, 42 □, 43 □, 44 □, 45 □, 46 □, 47 □, 48 □, 49 ■, 50 □
Papaveraceae *Corydalis govaniana* Wall. LI 34	Bhutyata	Herb	Roots	Powder	Skin burn	The powdered root is effective as antiperiodic, appetizer, diuretic and skin, tonic.	34	0.19	0.029	91.18	1 □, 2 □, 3 □, 4 □, 5 □, 6 □, 7 □, 8 □, 9 □, 10 □, 11 ■, 12 □, 13 □, 14 □, 15 □, 16 □, 17 □, 18 □, 19□, 20 □, 21□, 22 □, 23 □, 24 □, 25 □, 26 □, 27 □, 28 □, 29 □, 30 □, 31 □, 32 □, 33 □, 34 □, 35 □, 36 □, 37 □, 38 □, 39 □, 40 □, 41 □, 42 □, 43 □, 44 □, 45 □, 46 □, 47 □, 48 □, 49 □, 50 □
Phytolaceae *Phytolacea letsenia* L. LI 73	Amlok	Shrub	Flower, roots	Powder	Wound healing	Shade dried flowers are powdered and mixed with sugar, is recommended for wound healing.	37	0.21	0.027	83.78	1 □, 2 □, 3 □, 4 □, 5 □, 6 □, 7 □, 8 □, 9 □, 10 □, 11 □, 12 □, 13 □, 14 □, 15 □, 16 □, 17 □, 18 □, 19□, 20 □, 21□, 22 □, 23 □, 24 □, 25 □, 26 □, 27 □, 28 □, 29 □, 30 □, 31 □, 32 □, 33 □, 34 □, 35 □, 36 □, 37 □, 38 □, 39 □, 40 □, 41 □, 42 □, 43 □, 44 □, 45 □, 46 □, 47 □, 48 □, 49 □, 50 □
Pinaceae *Cedrus deodara* (Roxb. ex D.Don). LI 28	Deodar	Tree	Roots	Extracts	Skin problems	Oil extracted from roots is used for skin disorders.	36	0.20	0.028	86.11	1 □, 2 □, 3 □, 4 □, 5 □, 6 □, 7 □, 8 □, 9 □, 10 □, 11 □, 12 □, 13 □, 14 □, 15 □, 16 □, 17 □, 18 □, 19□, 20 □, 21□, 22 □, 23 □, 24 □, 25 □, 26 □, 27 □, 28 □, 29 □, 30 □, 31 □, 32 □, 33 □, 34 □, 35 □, 36 □, 37 □, 38 □, 39 □, 40 □, 41 □, 42 □, 43 □, 44 □, 45 □, 46 □, 47 □, 48 □, 49 □, 50 □
Pinaceae *Pinus roxburgii* Sarg LI 75/	Cheerh	Tree	Seed, stem	Juice	Skin problems	Juice of Seed is given 3 cups daily	16	0.09	0.063	56.25	1 □, 2 □, 3 □, 4 □, 5 □, 6 □, 7 □, 8 □, 9 □, 10 □, 11 □, 12 □, 13 □, 14 □, 15 □, 16 □, 17 □, 18 □, 19□, 20 □, 21□, 22 □, 23 □, 24 □, 25 □, 26 □, 27 □, 28 □, 29 □, 30 □, 31 □, 32 □, 33 □, 34 □, 35 □, 36 □, 37 □, 38 □, 39 □, 40 □, 41 □, 42 □, 43 □, 44 □, 45 □, 46 □, 47 □, 48 □, 49 □, 50 □
Pinaceae *Pinus wallichiana* A.B. Jacks. LI 76		Tree	Seed	Powder	Wound infection	The seeds are grinded to flour and few grains of sugar are mixed and taken with tea in the morning.	18	0.10	0.056	44.44	1 □, 2 □, 3 □, 4 □, 5 □, 6 □, 7 □, 8 □, 9 □, 10 □, 11 □, 12 □, 13 □, 14 □, 15 □, 16 □, 17 □, 18 □, 19□, 20 □, 21□, 22 □, 23 □, 24 □, 25 □, 26 □, 27 □, 28 □, 29 □, 30 □, 31 □, 32 □, 33 □, 34 □, 35 □, 36 □, 37 □, 38 □, 39 □, 40 □, 41 □, 42 □, 43 □, 44 □, 45 □, 46 □, 47 □, 48 □, 49 □, 50 □
Plantaginaceae *Picrorhiza kurrooa* Royle. ex Benth. LI 74	Kutakisafed	Herb	Roots		Burning sensations	It is useful in the treatment of burning sensation,	39	0.22	0.026	76.92	1 □, 2 □, 3 □, 4 □, 5 □, 6 □, 7 □, 8 □, 9 □, 10 □, 11 □, 12 □, 13 □, 14 □, 15 □, 16 □, 17 □, 18 □, 19□, 20 □, 21□, 22 □, 23 □, 24 □, 25 □, 26 □, 27 □, 28 □, 29 □, 30 □, 31 □, 32 □, 33 □, 34 □, 35 □, 36 □, 37 □, 38 □, 39 □, 40 □, 41 □, 42 □, 43 □, 44 □, 45 □, 46 □, 47 □, 48 □, 49 □, 50 □
Plantaginaceae *Plantago major* L*.* LI 78	Achar	Herb	Seed	Poultice	Skin problems, wound healing, boils	Polutice of fresh seeds is wrapped around the boils, after three day the pus drains out and the heals up within a week.	31	0.17	0.032	83.87	1 □, 2 □, 3 □, 4 □, 5 □, 6 □, 7 □, 8 □, 9 □, 10 □, 11 □, 12 □, 13 □, 14 □, 15 □, 16 □, 17 □, 18 □, 19□, 20 ■, 21□, 22 □, 23 □, 24 □, 25 □, 26 □, 27 □, 28 ■, 29 □, 30 □, 31 □, 32 □, 33 □□□, 34 □, 35 □, 36 □, 37 □, 38 □, 39 □, 40 □, 41 □, 42 □, 43 □, 44 □, 45 □, 46 □, 47 □, 48 □, 49 □, 50 □
Plantaginaceae *Plantago lanceolata* L. LI 79		Herb	Seed, Leaf	Poultice	Wounds healing	Leaf are applied to Wounds.	33	0.18	0.030	75.76	1 □, 2 □, 3 □, 4 □, 5 □, 6 □, 7 □, 8 □, 9 □, 10 □, 11 □, 12 □, 13 □, 14 □, 15 □, 16 □, 17 □, 18 □, 19□, 20 □, 21□, 22 □, 23 □, 24 □, 25 □, 26 □, 27 □, 28 □, 29 □, 30 □, 31 □, 32 □, 33 □, 34 □, 35 □, 36 □, 37 □, 38 □, 39 □, 40 □, 41 □, 42 □, 43 □, 44 □, 45 □, 46 □, 47 □, 48 □, 49 □, 50 □
Poaceae *Cynodon dactylon* (L.) Pers. LI 37	Kabalor	Herb	Whole plants	Powder	Wound healing, skin problems	Whole plant is grinded with water to cure skin problem	16	0.09	0.125	68.75	1 □, 2 □, 3 ■, 4 □, 5 ■, 6 □, 7 □, 8 □, 9 ●, 10 ■, 11 □, 12 □, 13 □, 14 □, 15 □, 16 □, 17 □, 18 □, 19□, 20 □, 21□, 22 □, 23 □, 24 □, 25 □, 26 □, 27 □, 28 □, 29 □, 30 □, 31 □, 32 □, 33 □, 34 ●, 35 □, 36 □, 37 □, 38 □, 39 □, 40 □, 41 □, 42 □, 43 □, 44 □, 45 □, 46 □, 47 □, 48 □, 49 □, 50 □
Polygonaceae *Fagopyrum acutatum* (Lehm.) Mansf. ex K.Hammer LI 45	Buck wheat	Herb	Leaf	Powder	Wound healing	Powder Leaf mixed with oil is applied over area	26	0.14	0.038	80.77	1 □, 2 □, 3 □, 4 □, 5 □, 6 □, 7 □, 8 □, 9 □, 10 □, 11 □, 12 □, 13 □, 14 □, 15 □, 16 □, 17 □, 18 □, 19□, 20 □, 21□, 22 □, 23 □, 24 □, 25 □, 26 □, 27 □, 28 □, 29 □, 30 □, 31 □, 32 □, 33 □, 34 □, 35 □, 36 □, 37 □, 38 □, 39 □, 40 □, 41 □, 42 □, 43 □, 44 □, 45 □, 46 □, 47 □, 48 □, 49 □, 50 □
Polygonaceae *Polygonum nepalense* Meissn. LI 81	Hulla	Herb	Leaf, Seeds	Paste	Wounds	A poultice prepared from the roots is used on fresh wounds.	30	0.17	0.033	76.67	1 □, 2 □, 3 □, 4 □, 5 □, 6 □, 7 □, 8 □, 9 □, 10 □, 11 □, 12 □, 13 □, 14 □, 15 □, 16 □, 17 □, 18 □, 19□, 20 □, 21□, 22 □, 23 □, 24 □, 25 □, 26 □, 27 □, 28 □, 29 □, 30 □, 31 □, 32 □, 33 □, 34 □, 35 □, 36 □, 37 □, 38 □, 39 □, 40 □, 41 □, 42 □, 43 □, 44 □, 45 □, 46 □, 47 □, 48 □, 49 □, 50 □
Polygonaceae *Rumex abyssinicus* Jacq. LI 87	Sa-shing		Roots	Decoction	Skin problem	Decoction of roots is taken with *Aloe vera* to treat skin problems	34	0.19	0.029	76.47	1 □, 2 □, 3 □, 4 □, 5 □, 6 □, 7 □, 8 □, 9 □, 10 □, 11 □, 12 □, 13 □, 14 □, 15 □, 16 □, 17 □, 18 □, 19□, 20 □, 21□, 22 □, 23 □, 24 □, 25 □, 26 □, 27 □, 28 □, 29 □, 30 □, 31 □, 32 □, 33 □, 34 □, 35 □, 36 □, 37 □, 38 □, 39 □, 40 □, 41 □, 42 □, 43 □, 44 □, 45 □, 46 □, 47 □, 48 □, 49 □, 50 □
Polygonaceae *Rumex dissectus* H. Lév. LI 88	Khatimmer	Herb	Leaf, roots	Extract, powder	Wound infections	Fresh Leaf extracts are crushed and used to stop wounds bleeding	29	0.16	0.034	86.21	1 □, 2 □, 3 □, 4 □, 5 □, 6 □, 7 □, 8 □, 9 □, 10 □, 11 □, 12 □, 13 □, 14 □, 15 □, 16 □, 17 □, 18 □, 19□, 20 □, 21□, 22 □, 23 □, 24 □, 25 □, 26 □, 27 □, 28 □, 29 □, 30 □, 31 □, 32 □, 33 □, 34 □, 35 □, 36 □, 37 □, 38 □, 39 □, 40 □, 41 □, 42 □, 43 □, 44 □, 45 □, 46 □, 47 □, 48 □, 49 □, 50 □
Polygonaceae *Rumex dentatus* L. LI 89	Shalkhay	Herbs	Leaf	Powder	Boils	2–3 leaves are powdered. Tea made by adding 4-5 grams of powder in 2 cups of water. This can be taken for treating boils.	27	0.15	0.037	88.89	1 □, 2 □, 3 □, 4 □, 5 □, 6 □, 7 □, 8 □, 9 □, 10 □, 11 □, 12 □, 13 □, 14 □, 15 □, 16 □, 17 □, 18 □, 19□, 20 □, 21□, 22 □, 23 □, 24 □, 25 □, 26 □, 27 □, 28 □, 29 □, 30 □, 31 □, 32 □, 33 □, 34 □, 35 □, 36 □, 37 □, 38 □, 39 □, 40 □, 41 □, 42 □, 43 □, 44 □, 45 □, 46 □, 47 □, 48 □, 49 □, 50 □
Polygonaceae *Fagopyrum tataricum* (L.) Gaertn. LI 46	Bro Kho-Bro	Herb	Leaf. seeds	Paste	Skin problem	Paste is applied on skin effected areas	35	0.19	0.029	91.43	1 □, 2 □, 3 □, 4 □, 5 □, 6 □, 7 □, 8 □, 9 □, 10 □, 11 □, 12 □, 13 □, 14 □, 15 □, 16 □, 17 □, 18 □, 19□, 20 □, 21□, 22 □, 23 □, 24 □, 25 □, 26 □, 27 □, 28 □, 29 □, 30 □, 31 □, 32 □, 33 □, 34 □, 35 □, 36 □, 37 □, 38 □, 39 □, 40 □, 41 □, 42 □, 43 □, 44 □, 45 □, 46 □, 47 □, 48 □, 49 □, 50 □
Primulaceae *Androsace rotundifolia* Lehm. ex Roem. & Schult. LI 9	Marcholla	Herb	Leaf	Extracts	Skin problem	Aqueous leaf extract is prepared and used in treating skin infections.	22	0.12	0.045	72.73	1 □, 2 □, 3 □, 4 □, 5 □, 6 □, 7 □, 8 □, 9 □, 10 □, 11 □, 12 □, 13 □, 14 □, 15 □, 16 □, 17 □, 18 □, 19□, 20 □, 21□, 22 □, 23 □, 24 □, 25 □, 26 □, 27 □, 28 □, 29 □, 30 □, 31 □, 32 □, 33 □, 34 □, 35 □, 36 □, 37 □, 38 □, 39 □, 40 □, 41 □, 42 □, 43 □, 44 □, 45 □, 46 □, 47 □, 48 □, 49 □, 50 □
Pteridaceae *Adiantum venustum* D. Don LI 4	Pata, kakwa	Herb	Leaf	Paste	Wound healing	The rhizome paste is applied to heal cuts and wounds.	48	0.27	0.021	91.67	1 □, 2 □, 3 □, 4 □, 5 □, 6 □, 7 □, 8 □, 9 □, 10 □, 11 □, 12 □, 13 □, 14 □, 15 □, 16 □, 17 □, 18 □, 19□, 20□, 21□, 22 □, 23 □, 24 □, 25 □, 26 □, 27 □, 28 □, 29 □, 30 □, 31 □, 32 □, 33 □, 34 □, 35 □, 36 □, 37 □, 38 □, 39 □, 40 □, 41 □, 42 □, 43 □, 44 □, 45 □, 46 □, 47 □, 48 □, 49 □, 50 □
Ranunculaceae *Aconitum chasmanthum* Stapf ex Holmes LI 2	Bishmoulo (Shina) Mori	Herb	Leaf	Decoction	Mumps, measles	Decoction of the Leaf are given for 2 weeks to cure diseases	44	0.24	0.023	88.64	1 □, 2 □, 3 □, 4 □, 5 □, 6 □, 7 □, 8 □, 9 □, 10 □, 11 □, 12 □, 13 □, 14 □, 15 □, 16 □, 17 □, 18 □, 19□, 20 □, 21□, 22 □, 23 □, 24 □, 25 □, 26 □, 27 □, 28 □, 29 □, 30 □, 31 □, 32 □, 33 □, 34 □, 35 □, 36 □, 37 □, 38 □, 39 □, 40 □, 41 □, 42 □, 43 □, 44 □, 45 □, 46 □, 47 □, 48 □, 49 □, 50 □
Ranunculaceae *Aconitum delphinifolium* DC. LI 3	Booma	Herb	Leaf	Decoction	Wound healing, boils	Dried leaves are boiled in water to make decoction and is taken on daily basis to cure boils.	31	0.17	0.065	90.32	1 □, 2 □, 3 □, 4 □, 5 □, 6 □, 7 □, 8 □, 9 □, 10 □, 11 □, 12 □, 13 □, 14 □, 15 □, 16 □, 17 □, 18 □, 19□, 20 □, 21□, 22 □, 23 □, 24 □, 25 □, 26 □, 27 □, 28 □, 29 □, 30 □, 31 □, 32 □, 33 □, 34 □, 35 □, 36 □, 37 □, 38 □, 39 □, 40 □, 41 □, 42 □, 43 □, 44 □, 45 □, 46 □, 47 □, 48 □, 49 □, 50 □
Ranunculaceae *Aquilegia pubiflora* Wall. ex Royle LI 13	Koo-kuk	Herb	Leaf, floral parts	Paste	Skin burns and wound healing	Fresh plant parts are crushed in water to prepare paste and applied on affected areas to avoid pain from burns and wounds.	39	0.22	0.051	79.49	1 □, 2 □, 3 □, 4 □, 5 □, 6 □, 7 □, 8 □, 9 ■, 10 □, 11 □, 12 □, 13 □, 14 □, 15 □, 16 □, 17 □, 18 □, 19□, 20 □, 21□, 22 □, 23 □, 24 □, 25 □, 26 □, 27 □, 28 □, 29 □, 30 □, 31 □, 32 □, 33 ■, 34 □, 35 □, 36 □, 37 □, 38 □, 39 □, 40 □, 41 □, 42 □, 43 □, 44 □, 45 □, 46 □, 47 □, 48 □, 49 □, 50 □
Ranunculaceae *Caltha alba* Cambess LI 25**/**	Neel kanth		Leaf	Extract	Skin problems	Leaf extract is used for cleaning skin lesions, sores and skin diseases.	21	0.12	0.048	80.95	1 □, 2 □, 3 □, 4 □, 5 □, 6 □, 7 □, 8 □, 9 □, 10 □, 11 □, 12 □, 13 □, 14 □, 15 □, 16 □, 17 □, 18 □, 19□, 20 □, 21□, 22 □, 23 □, 24 □, 25 □, 26 □, 27 □, 28 □, 29 □, 30 □, 31 □, 32 □, 33 □, 34 □, 35 □, 36 □, 37 □, 38 □, 39 □, 40 □, 41 □, 42 □, 43 □, 44 □, 45 □, 46 □, 47 □, 48 □, 49 □, 50 □
Ranunculaceae *Nigella sativa* L. LI 69	Kaloongee	Herb	Seed, Leaf		Wound healing	Latex is effective for rheumatic pain.	26	0.14	0.038	61.54	1 ■, 2 □, 3 □, 4 □, 5 □, 6 □, 7 □, 8 □, 9 □, 10 □, 11 □, 12 □, 13 □, 14 □, 15 ■, 16 □, 17 □, 18 □, 19□, 20 □, 21□, 22 □, 23 □, 24 □, 25 □, 26 □, 27 □, 28 □, 29 □, 30 □, 31 □, 32 □, 33 □, 34 □, 35 □, 36 □, 37 □, 38 □, 39 □, 40 □, 41 □, 42 □, 43 □, 44 □, 45 □, 46 □, 47 □, 48 □, 49 □, 50 □
Rhamnaceae *Colubrina oppositifolia* Brongn. ex H. Mann LI 23	Lansa	Shrub	Leaf	Paste	Wound healing, Skin problem	Leaf Paste are applied on wound and bruises	32	0.18	0.063	81.25	1 □, 2 □, 3 □, 4 □, 5 □, 6 □, 7 □, 8 □, 9 □, 10 □, 11 □, 12 □, 13 □, 14 □, 15 □, 16 □, 17 □, 18 □, 19□, 20 □, 21□, 22 □, 23 □, 24 □, 25 □, 26 □, 27 □, 28 □, 29 □, 30 □, 31 □, 32 □, 33 □, 34 □, 35 □, 36 □, 37 □, 38 □, 39 □, 40 □, 41 □, 42 □, 43 □, 44 □, 45 □, 46 □, 47 □, 48 □, 49 □, 50 □
Rosaceae *Malus pumila* Mill LI 64	Manra	Tree	Leaf	Raw, Juice	Boils	Juice extracted from the Leafare used in boils	28	0.16	0.071	75.00	1 □, 2 □, 3 □, 4 □, 5 □, 6 □, 7 □, 8 □, 9 □, 10 □, 11 □, 12 □, 13 □, 14 □, 15 □, 16 □, 17 □, 18 □, 19□, 20 □, 21□, 22 □, 23 □, 24 □, 25 □, 26 □, 27 □, 28 □, 29 □, 30 □, 31 □, 32 □, 33 □, 34 □, 35 □, 36 □, 37 □, 38 □, 39 □, 40 □, 41 □, 42 □, 43 □, 44 □, 45 □, 46 □, 47 □, 48 □, 49 □, 50 □
Rosaceae *Prunus armeniaca* L. LI 82	Apricot	Tree	Fruit		Skin problem		32	0.18	0.031	96.88	1 □, 2 □, 3 □, 4 □, 5 ■, 6 □, 7 □, 8 □, 9 □, 10 □, 11 □, 12 □, 13 □, 14 □, 15 □, 16 □, 17 □, 18 □, 19□, 20 □, 21□, 22 □, 23 □, 24 □, 25 □, 26 □, 27 □, 28 □, 29 □, 30 □, 31 □, 32 □, 33 ●, 34 □, 35 □, 36 □, 37 □, 38 □, 39 □, 40 □, 41 □, 42 □, 43 □, 44 □, 45 □, 46 □, 47 □, 48 □, 49 □, 50 □
Rosaceae *Prunus persica* (L.) Batsch LI 83	Aru	Tree	Fruit and Leaf		Skin problems		18	0.10	0.056	55.56	1 □, 2 □, 3 □, 4 □, 5 ■, 6 □, 7 □, 8 □, 9 □, 10 □, 11 □, 12 □, 13 □, 14 □, 15 □, 16 □, 17 □, 18 □, 19□, 20 □, 21□, 22 □, 23 □, 24 □, 25 □, 26 □, 27 □, 28 □, 29 □, 30 □, 31 □, 32 □, 33 □, 34 □, 35 □, 36 □, 37 □, 38 □, 39 □, 40 □, 41 □, 42 □, 43 □, 44 □, 45 □, 46 ●, 47 □, 48 □, 49 □, 50 □
Rosaceae *Rosa chinensis* Jacq LI 85	Gulab	Shrub	Flower	Raw	Skin problem	Fruit is used to reduce pain	40	0.22	0.050	95.00	1 □, 2 □, 3 □, 4 □, 5 □, 6 □, 7 □, 8 □, 9 □, 10 □, 11 □, 12 □, 13 □, 14 □, 15 □, 16 □, 17 □, 18 □, 19□, 20 □, 21□, 22 □, 23 □, 24 □, 25 □, 26 □, 27 □, 28 □, 29 □, 30 □, 31 □, 32 □, 33 □, 34 □, 35 □, 36 □, 37 □, 38 □, 39 □, 40 □, 41 □, 42 □, 43 □, 44 □, 45 □, 46 □, 47 □, 48 □, 49 □, 50 □
Rosaceae *Rubus abchaziensis* Sudre LI 86	Akhray, Karwarra	Shrub	Flowers, roots	Decoction	Wound healing, boils	Fruit decoction is given for 2 week to cure wounds and boils.	36	0.20	0.028	75.00	1 □, 2 □, 3 □, 4 □, 5 □, 6 □, 7 □, 8 □, 9 □, 10 □, 11 □, 12 □, 13 □, 14 □, 15 □, 16 □, 17 □, 18 □, 19□, 20 □, 21□, 22 □, 23 □, 24 □, 25 □, 26 □, 27 □, 28 □, 29 □, 30 □, 31 □, 32 □, 33 □, 34 □, 35 □, 36 □, 37 □, 38 □, 39 □, 40 □, 41 □, 42 □, 43 □, 44 □, 45 □, 46 □, 47 □, 48 □, 49 □, 50 □
Rubiaceae *Galium abaujense* Borbás LI 48	Khrrhatani	Herb	Leaf	Poultice	Wound healing	Poultice prepared from leaves is applied on wounds and used as an antiseptic.	19	0.11	0.053	36.84	1 □, 2 □, 3 □, 4 □, 5 □, 6 □, 7 □, 8 □, 9 □, 10 □, 11 □, 12 □, 13 □, 14 □, 15 □, 16 □, 17 □, 18 □, 19□, 20 □, 21□, 22 □, 23 □, 24 □, 25 □, 26 □, 27 □, 28 □, 29 □, 30 □, 31 □, 32 □, 33 □, 34 □, 35 □, 36 □, 37 □, 38 □, 39 □, 40 □, 41 □, 42 □, 43 □, 44 □, 45 □, 46 □, 47 □, 48 □, 49 □, 50 □
Rubiaceae *Gallium aparine* L*.* LI 49	Loothar	Herb	Leaf	Poultice	Wound healing	Leaf are externally used on wounds as antiseptic	21	0.12	0.048	80.95	1 □, 2 □, 3 □, 4 □, 5 □, 6 □, 7 □, 8 □, 9 □, 10 □, 11 □, 12 □, 13 □, 14 □, 15 □, 16 □, 17 □, 18 □, 19□, 20 □, 21□, 22 □, 23 □, 24 □, 25 □, 26 □, 27 □, 28 □, 29 □, 30 □, 31 □, 32 □, 33 □, 34 □, 35 □, 36 □, 37 □, 38 □, 39 □, 40 □, 41 □, 42 □, 43 □, 44 □, 45 □, 46 □, 47 □, 48 □, 49 □, 50 □
Rutaceae *Zanthoxylum armatum* DC LI 11	Dumbara	Shrubs	Leaf	Raw, paste	Skin burn	Fresh Leaf paste are used to cure skin burn	19	0.11	0.053	57.89	1 □, 2 □, 3 □, 4 □, 5 ●, 6 □, 7 □, 8 □, 9 □, 10 □, 11 □, 12 □, 13 □, 14 □, 15 □, 16 □, 17 □, 18 □, 19□, 20 □, 21□, 22 □, 23 □, 24 □, 25 □, 26 □, 27 □, 28 □, 29 □, 30 □, 31 □, 32 □, 33 □, 34 □, 35 □, 36 □, 37 □, 38 □, 39 □, 40 □, 41 □, 42 □, 43 □, 44 □, 45 □, 46 □, 47 □, 48 □, 49 □, 50 □
Rutaceae *Citrus medica* L. LI 30	Lemmon	Tree	Fruit	Juice	skin irritation	Juice of fruit is applied on skin to reduce skin irritation	14	0.08	0.071	78.57	1 □, 2 □, 3 ●, 4 □, 5 □, 6 □, 7 □, 8 □, 9 □, 10 □, 11 □, 12 □, 13 □, 14 □, 15 □, 16 □, 17 □, 18 □, 19□, 20 □, 21□, 22 □, 23 □, 24 □, 25 □, 26 □, 27 □, 28 □, 29 □, 30 □, 31 □, 32 □, 33 □, 34 □, 35 □, 36 □, 37 □, 38 □, 39 □, 40 □, 41 □, 42 □, 43 □, 44 □, 45 □, 46 □, 47 □, 48 □, 49 □, 50 □
Rutaceae *Citrus sinensis* L. LI 31	Orange	Tree	Fruit	Raw	Pimples	Fruit as a whole is used to reduce pimples	20	0.11	0.050	80.00	1 ■, 2 □, 3 □, 4 □, 5 □, 6 □, 7 □, 8 □, 9 □, 10 □, 11 □, 12 □, 13 □, 14 □, 15 □, 16 □, 17 □, 18 □, 19□, 20 □, 21□, 22 □, 23 □, 24 □, 25 □, 26 □, 27 □, 28 □, 29 □, 30 □, 31 □, 32 □, 33 □, 34 □, 35 □, 36 □, 37 □, 38 □, 39 □, 40 □, 41 □, 42 □, 43 □, 44 □, 45 □, 46 □, 47 □, 48 □, 49 ●, 50 □
Salicaceae *Salix babylonica* L. LI 91	Bainsa	Tree	Leaf, roots	Extract	Skin cleanser	The extract of Leaf and root are taken for skin cleanser	20	0.11	0.100	60.00	1 □, 2 □, 3 □, 4 □, 5 □, 6 □, 7 □, 8 □, 9 □, 10 □, 11 □, 12 □, 13 □, 14 □, 15 □, 16 □, 17 □, 18 □, 19□, 20 □, 21□, 22 □, 23 □, 24 □, 25 □, 26 □, 27 □, 28 □, 29 □, 30 □, 31 □, 32 □, 33 □, 34 □, 35 □, 36 □, 37 □, 38 □, 39 □, 40 □, 41 □, 42 □, 43 □, 44 □, 45 □, 46 □, 47 □, 48 □, 49 □, 50 □
Sapindaceae *Dodonaea viscosa* (L.) Jacq LI 42	Ghwaraskay, Santha	Shrub	Leaf	Powders	Skin burn, wound healing	Grinded leaves are mixed in water to make juice and used for skin problems.	33	0.18	0.061	84.85	1 □, 2 □, 3 □, 4 □, 5 ■, 6 □, 7 □, 8 □, 9 □, 10 □, 11 □, 12 □, 13 □, 14 □, 15 □, 16 □, 17 □, 18 □, 19□, 20 □, 21□, 22 □, 23 □, 24 □, 25 □, 26 □, 27 □, 28 □, 29 □, 30 □, 31 □, 32 □, 33 □, 34 □, 35 □, 36 □, 37 □, 38 □, 39 □, 40 □, 41 □, 42 □, 43 □, 44 □, 45 □, 46 □, 47 □, 48 □, 49 □, 50 □
Saxifragaceae *Bergenia ciliata* (Haw.) Sternb LI 16	Batweyaa		Bark	Paste	Wound healing	Paste of Bark is antibacterial and is used to heal up wounds and cuts.	18	0.10	0.056	61.11	1 □, 2 □, 3 □, 4 □, 5 ■, 6 □, 7 □, 8 □, 9 □, 10 □, 11 □, 12 □, 13 □, 14 □, 15 □, 16 □, 17 □, 18 □, 19□, 20 ■, 21□, 22 □, 23 □, 24 □, 25 □, 26 □, 27 □, 28 □, 29 □, 30 □, 31 □, 32 □, 33 □, 34 □, 35 □, 36 □, 37 □, 38 □, 39 □, 40 □, 41 □, 42 □, 43 □, 44 □, 45 □, 46 □, 47 □, 48 □, 49 □, 50 □
Saxifragaceae *Bergenia ligulata* Engl. LI 17	ZakamJat	Herb	Whole plant	Extracts	Wound healing, boil	Extract of whole dried plant is mixed in hot water and applied externally on, boil, cuts and wounds.	17	0.09	0.118	76.47	1 □, 2 □, 3 □, 4 □, 5 □, 6 □, 7 □, 8 □, 9 □, 10 □, 11 □, 12 □, 13 □, 14 □, 15 □, 16 □, 17 □, 18 □, 19□, 20 □, 21□, 22 □, 23 □, 24 □, 25 □, 26 □, 27 □, 28 □, 29 □, 30 □, 31 □, 32 □, 33 □, 34 □, 35 □, 36 □, 37 □, 38 □, 39 □, 40 □, 41 □, 42 □, 43 □, 44 □, 45 □, 46 □, 47 □, 48 □, 49 □, 50 □
Saxifragaceae *Bergenia stracheyi* Hook.f. & Thomson) Engl LI 18	Zakham-i- hayat	Herb	Leaf, flower	Powder	Sun strokes, wound healing	Powder of Leaf and flowers are mixed with butter and sun blocking cream.	34	0.19	0.059	85.29	1 □, 2 □, 3 □, 4 □, 5 □, 6 □, 7 □, 8 □, 9 □, 10 □, 11 □, 12 □, 13 □, 14 □, 15 □, 16 □, 17 □, 18 □, 19□, 20 □, 21□, 22 □, 23 □, 24 □, 25 □, 26 □, 27 □, 28 ●, 29 □, 30 □, 31 □, 32 □, 33 □, 34 □, 35 □, 36 □, 37 □, 38 □, 39 □, 40 □, 41 □, 42 □, 43 □, 44 □, 45 □, 46 □, 47 □, 48 □, 49 □, 50 □
Scrophulariaceae *Verbascum thapsus* L. LI 106	Gadikand		Aerial part	Infusion	Pimples, skin problem	Aerial plants are crushed, mixed in water and taken for 4–5 days to cure skin problems.	38	0.21	0.053	76.32	1 □, 2 □, 3 □, 4 □, 5 □, 6 □, 7 □, 8 □, 9 □, 10 □, 11 □, 12 □, 13 □, 14 □, 15 □, 16 □, 17 □, 18 □, 19□, 20 □, 21□, 22 □, 23 □, 24 □, 25 □, 26 □, 27 □, 28 □, 29 □, 30 □, 31 □, 32 □, 33 □, 34 □, 35 □, 36 □, 37 □, 38 □, 39 □, 40 □, 41 □, 42 □, 43 □, 44 □, 45 □, 46 □, 47 □, 48 □, 49 □, 50 □
Solanaceae *Datura stramonium* L. LI 40	Dhatura	Shrub	Seeds, Leaf	Paste	Boils	Leaf are applied on boils	21	0.12	0.048	71.43	1 □, 2 □, 3 ●, 4 ●, 5 ■, 6 □, 7 ●, 8 □, 9 □, 10 □, 11 □, 12 □, 13 □, 14 □, 15 □, 16 □, 17 □, 18 □, 19□, 20 □, 21□, 22 □, 23 □, 24 □, 25 □, 26 □, 27 □, 28 □, 29 □, 30 □, 31 □, 32 □, 33 □, 34 □, 35 □, 36 □, 37 □, 38 □, 39 □, 40 □, 41 □, 42 □, 43 □, 44 □, 45 □, 46 □, 47 □, 48 □, 49 □, 50 □
Solanaceae *Solanum virginianum* L. LI 95	Kandiari		Fruits, Leaf	Decoction, extract	Skin problem, swelling of skin	Fruits are boiled and prepared decoction mixed in water is used for taking bath to cure skin problems, The fruits and leaves extract are applied on body swellings to get relief.	28	0.16	0.036	96.43	1 □, 2 □, 3 □, 4 □, 5 □, 6 □, 7 □, 8 □, 9 □, 10 □, 11 □, 12 □, 13 □, 14 □, 15 □, 16 □, 17 □, 18 □, 19□, 20 □, 21□, 22 □, 23 □, 24 □, 25 □, 26 □, 27 □, 28 □, 29 □, 30 □, 31 □, 32 □, 33 □, 34 □, 35 □, 36 □, 37 □, 38 □, 39 □, 40 □, 41 □, 42 □, 43 □, 44 □, 45 □, 46 □, 47 □, 48 □, 49 □, 50 □
Tamaricaceae *Tamarix aphylla* (L.) H. Karst. LI 98	Ghaz	Herb	Leaf	Decoction	Wounds	The decoction of the plant is given to the patient for 1 week	12	0.07	0.083	58.33	1 □, 2 □, 3 □, 4 □, 5 □, 6 □, 7 □, 8 □, 9 □, 10 □, 11 □, 12 □, 13 □, 14 □, 15 □, 16 □, 17 □, 18 □, 19□, 20 □, 21□, 22 □, 23 □, 24 □, 25 □, 26 □, 27 □, 28 □, 29 □, 30 □, 31 □, 32 □, 33 □, 34 □, 35 □, 36 □, 37 □, 38 □, 39 □, 40 □, 41 □, 42 □, 43 □, 44 □, 45 □, 46 □, 47 □, 48 □, 49 □, 50 □
Taxaceae *Taxus wallichiana* Zucc. LI 100	Bermi		Fruits	Extracts	Skin problems	Extract of the fruits obtained and is used daily	29	0.16	0.034	72.41	1 □, 2 □, 3 □, 4 □, 5 □, 6 □, 7 □, 8 □, 9 □, 10 □, 11 □, 12 □, 13 □, 14 □, 15 □, 16 □, 17 □, 18 □, 19□, 20 □, 21□, 22 □, 23 □, 24 □, 25 □, 26 □, 27 □, 28 □, 29 □, 30 □, 31 □, 32 □, 33 □, 34 □, 35 □, 36 □, 37 □, 38 □, 39 □, 40 □, 41 □, 42 □, 43 □, 44 □, 45 □, 46 □, 47 □, 48 □, 49 □, 50 □
Thymelaeaceae *Daphne mucronata S* Royle LI 39		Shrub	Seeds	Raw	Skin problem	Seeds can be used for skin diseases.	39	0.22	0.026	74.36	1 □, 2 □, 3 □, 4 □, 5 □, 6 □, 7 □, 8 □, 9 □, 10 □, 11 □, 12 □, 13 □, 14 □, 15 □, 16 □, 17 □, 18 □, 19□, 20 □, 21□, 22 ■, 23 □, 24 □, 25 □, 26 □, 27 □, 28 □, 29 □, 30 □, 31 □, 32 □, 33 □, 34 □, 35 □, 36 □, 37 ■, 38 □, 39 □, 40 □, 41 □, 42 □, 43 □, 44 □, 45 □, 46 □, 47 □, 48 □, 49 □, 50 □
Urticaceae *Urtica dioica* L. LI 104	Bichu- buti	Herb	Leaf, Seeds	Paste	Wound healing	Its Leaf and seeds are mixed with oil and used on skin for wound.	18	0.10	0.056	83.33	1 □, 2 □, 3 □, 4 □, 5 □, 6 □, 7 □, 8 □, 9 □, 10 □, 11 □, 12 ■, 13 □, 14 □, 15 □, 16 □, 17 □, 18 □, 19□, 20 □, 21□, 22 ■, 23 □, 24 □, 25 □, 26 □, 27 □, 28 ■, 29 □, 30 □, 31 □, 32 □, 33 □, 34 □, 35 □, 36 □, 37 □, 38 □, 39 □, 40 □, 41 □, 42 □, 43 □, 44 □, 45 □, 46 □, 47 □, 48 □, 49 □, 50 □

*FC* Frequency of citation, *RFC* Relative frequency of citation, *UV* Used value, *FL* Fidelity level, □ = Dissimilar plants with previous literature, ■ = Similar plants with previous literature; ● Dissimilar plants with previous literature

1 = [47], 2 = [48], 3 = [22], 4 = [29], 5 = [1], 6 = [3], 7= [49], 8 = [50], 9 = [51], 10 = [26]. 11 = [7], 12 = [27], 13 = [5], 14 = [52], 15 = [53], 16 = [54]. 17 = [55], 18 = [28], 19 = [56], 20 = [57], 21 = [58], 22 = [59], 23 = [60], 24 = [61], 25 = [17], 26 = [62], 27 = [63], 28 = [64], 29 = [65], 30 = [66], 31 = [67], 32 = [28], 33 = [68], 34 = [69], 35 = [30], 36 = [70], 37 = [71], 38 = [72], 39 = [73], 40 = [11], 41 = [74], 42 = [75], 43 = [76], 44 = [77], 45 = [78], 46 = [79], 47 = [80], 48 = [81], 49 = [82] 50 = [83].

### Plant parts used in herbal medicines

**Fig. 3:**
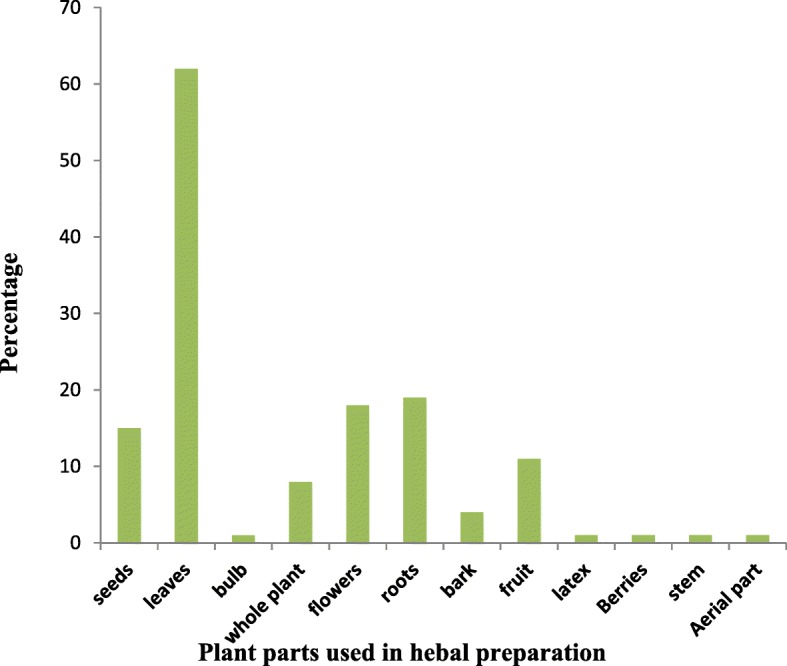
Medicinal plant parts utilized for skin disorders in Northern Pakistan

Leaves (62%) were reported to be the most frequently used plant part to prepare herbal medicine either by singly or mixes by other plant parts. Leaves were followed by roots (19 species), flowers (18 species), seeds (15 species), fruit (11 species), whole plant (8 species) and stem, bulb, latex, aerial parts contributed (1 species each) (Fig.3). A schematic representation of part used of medicinal plants is shown in (Additional file 1).

### Mode of preparation

**Fig. 4:**
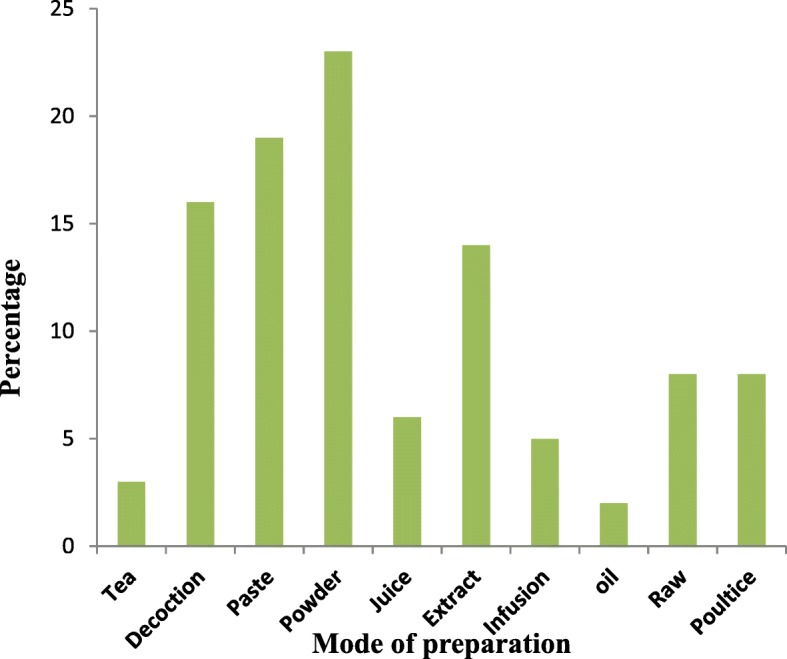
Mode of utilization of medicinal plants used for skin disorders in Northern Pakistan

Mode of administration for herbal remedies used for treating skin diseases include decoction, infusion, powder, poultice, raw, extract, juice, cooked, paste and oil. Among various preparation methods, the powder was the most frequently used (23 species), followed by paste (19 species), decoction (16 species), extract (14 species), raw and poultice (each has 8 species) (Fig. 4). A schematic representation of the mode of utilization of medicinal plants is shown in (Additional file 1).

### Used categories in skin diseases

**Fig. 5:**
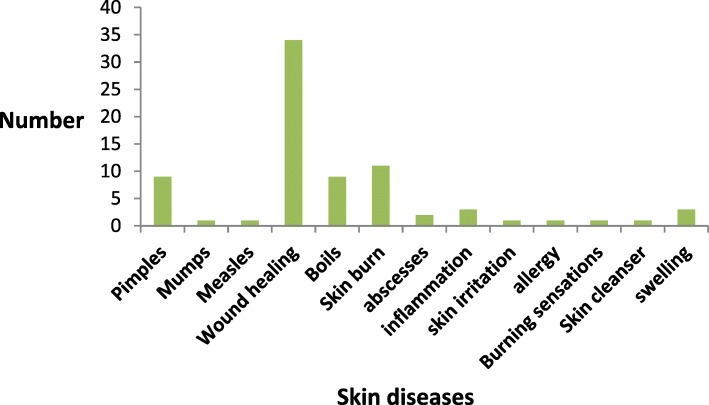
Categories diseases of medicinal plants used for skin disorders in Northern Pakistan

In this study, the skin diseases were assembled into 13 groups. The skin category includes pimples, mumps, measles, wound healing, boils, skin burns, abscesses, inflammation, skin irritation, allergy, burning sensation, skin cleanser and sensation (Table 2). In this study, the maximum figure of plant was used in handling for wound healing (34 species) followed by skin burn (11 species). Other important skin ailments treated by plant flora in the area were boils and pimples (9 species). The lowest citation reports (1%) were recorded for mumps, measles and skin irritations (Fig. 5).

### Quantitative ethnobotany

#### Value of medicinal plant

In addition to the use of questionnaires, various analytical tools were required so it could be possible to do quantification of data by cross verification of indigenous information to treat skin diseases in the study site. Species with the highest use value was *Pisum sativum* (Fabaceae) (UV 0.143) (Table 2). Other important plants were *Cynodon dactylon* (UV 0.125) reported by 16 participants and *Bergenia ligulata* reported by 17 participants (UV 0.118) (Table 2). *Adiantum venustum* had very low use value (UV 0.021).

#### Relative frequency of citation (RFC %)

The RFC represented the prominent species used for skin related diseases based on the ratio between the number of participants (FC) for a plants and the overall number of participants in the research study. RFC ranged from 0.07 to 0.25 and we classified all species into 3 groups: RFC 0.07 to 0.12 (39 species); RFC, 0.13 to 0.18 (37 species); RFC 0.19 to 0.27 (30 species) (Table 2). According to pharmacological and ethnobotanical records, the majority of plants in the first group were reported with high medicinal potential. The highest values were recorded for *Adiantum venustum* (0.27) used in the form of paste for wound healing properties, *Artemisia fragrans* (0.25) used in the treatment of boils, similarly *Aconitum chasmanthum* (0.24) used as a decoction for treatment of mumps and measles. Other high RFC species were *Trigonella foenum-graecum, Verbascum thapsus, Saussurea heteromala, Rosa chinensis*, *Gerbera gossypina*, *Helianthus annuus* and *Aquilegia pubiflora*.

#### Fidelity level (FL)

FL value is calculated for handling specific ailment in this study site. We examined the disease categories to focus the most significant medicinal plant species in each category of skin ailment in terms of FL. It is analyzed for the plant species which were used to cure the most commonly reported category for high FL values 100% and lower FL value 36.8%. FL values were classified into four FL classes (Table 2). FL value of class one was 100% (2 species), class two 97 to 89% (18 species), class three 88 to 79% (44 species), class four 78 to 69% (31 species), class five 68 to 33% (11 species). In the present study, *Salix babylonica* and *Sonchus asper* had an FL of 100%, *Prunus armeniaca* 96.8%, and *Momordica charantia* 94.74%. Lowest values were found for *Pinus wallichiana* (44.4) and *Galium abaujense* (36.8).

#### Family importance value (FIV)

**Fig. 6:**
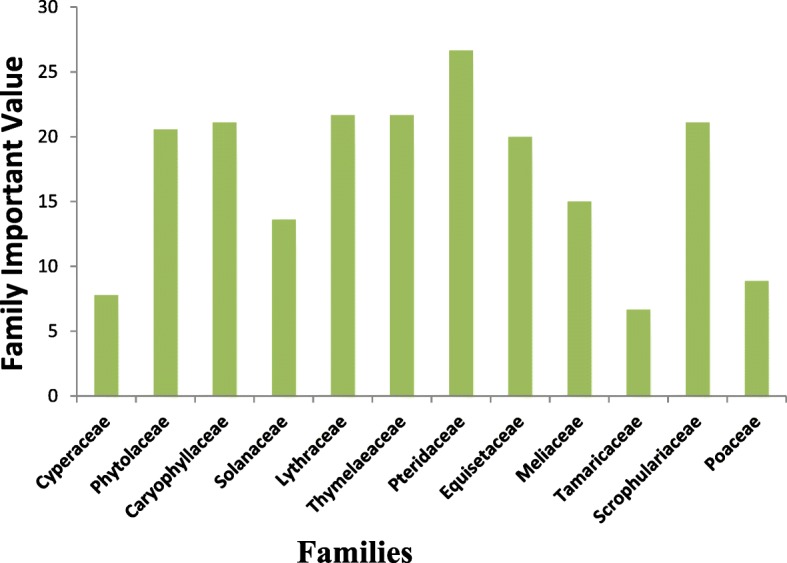
Family importance value of medicinal plants utilized for skin disorders in Northern Pakistan

The analysis of family importance value reported to Pteridaceae has the maximum FIV (26.6%), followed by Fabaceae (22.2%), Scrophulariaceae, Thymelaeaceae and Caryophyllaceae (21.6). Lowest values were observed for Cyperaceae 7.7 (Fig.6). These medicinal plants are explored equally by all the communities on a regular basis and the folk knowledge is constant.

#### Jaccard index (JI)

A comparison of medicinal uses of plants was made by analyzing 50 research papers from aligned countries (Table 2). The review of the literature showed that 106 reported medicinal plant species share similar uses fluctuated from 0% [29] to 13.2% while nonsimilar usage from 3.77 [64] to 0% [70]. The lowest degree of similarity was found in the studies reported in India and South Africa on skin diseases by [5, 49–51] (Table 3). The comparison was based on skin disease reports in several studies, presenting the usage of therapeutic plants for the cure of skin infections in local communities.

**Table 3:** Comparison of the present study with previous literature at local, regional and global level

S. No	Study Site	Year	Number of plant spp. recorded in aligned areas	Plants reported for similar uses	Plants reported for dissimilar uses	Total plant spp. common in both the area	%age of plant spp. common in both the areas	Plant species enlisted only in aligned areas	Species enlisted only in study area	%age of plant spp. enlisted only in the study area	%age of plant species with similar uses	%age of plant species with dissimilar uses	Jaccard index (JI)	Citation
1	Amman, Jordan	2003	58	6	1	7	12.07	51	99	93.40	5.6603774	0.94	4.90	[47]
2	Karnataka, India	2003	31	0	1	1	3.23	30	105	99.06	0	0.94	0.75	[48]
3	Assamese, India	2006	85	5	2	7	8.24	78	99	93.40	4.7169811	1.89	4.12	[22]
4	Central Kenya	2007	57	0	1	1	1.75	56	105	99.06	0	0.94	0.63	[29]
5	North-West Frontier Province, Pakistan	2010	66	14	1	15	22.73	51	91	85.85	13.207547	0.94	11.81	[1]
6	Central Chaco, Argentina	2010	72	1	1	2	2.78	70	104	98.11	0.9433962	0.94	1.16	[3]
7	South Africa	2014	117	1	1	2	1.71	115	104	98.11	0.9433962	0.94	0.92	[49]
8	Eastern Cape, South Africa	2014	106	2	1	3	2.83	103	103	97.17	1.8867925	0.94	1.48	[50]
9	Uttarakhand, India	2014	90	5	3	8	8.89	82	98	92.45	4.7169811	2.83	4.65	[51]
10	Pakistan	2013	50	3	1	4	8.00	46	102	96.23	2.8301887	0.94	2.78	[26]
11	France	2015	1	1	0	1	100.00	0	105	99.06	0.9433962	0.00	0.96	[7]
12	Kenya	2015	25	1	0	1	4.00	24	105	99.06	0.9433962	0.00	0.78	[27]
13	South Africa	2013	47	0	0	0	0.00	47	106	100.00	0	0.00	0.00	[5]
14	India	1992	50	0	0	0	0.00	50	106	100.00	0	0.00	0.00	[52]
15	North West Punjab, Pakistan	2012	12	3	0	3	25.00	9	103	97.17	2.8301887	0.00	2.75	[53]
16	Saudi Arabia	2015	4	0	0	0	0.00	4	106	100.00	0	0.00	0.00	[54]
17	India		95	2	1	3	3.16	92	103	97.17	1.8867925	0.94	1.56	[55]
18	Nigeria	2008	41	1	1	2	4.88	39	104	98.11	0.9433962	0.94	1.42	[28]
19	India	2010	11	0	0	0	0.00	11	106	100.00	0	0.00	0.00	[84]
20	South Africa	1999	9	3	1	4	44.44	5	102	96.23	2.8301887	0.94	3.88	[57]
21	Eastern Cape, South Africa	2016	1	0	0	0	0.00	1	106	100.00	0	0.00	0.00	[58]
22	Iran	2014	18	3	1	4	22.22	14	102	96.23	2.8301887	0.94	3.57	[59]
23	Haryan, India	2012	100	0	0	0	0.00	100	106	100.00	0	0.00	0.00	[60]
24	India	2012	1	0	0	0	0.00	1	106	100.00	0	0.00	0.00	
25	Thailand	2015	55	0	0	0	0.00	55	106	100.00	0	0.00	0.00	[17]
26	Mizoram, India	2014	4	0	0	0	0.00	4	106	100.00	0	0.00	0.00	[62]
27	Peru, Amercia	1997	9	0	0	0	0.00	9	106	100.00	0	0.00	0.00	[63]
28	Palestine, Israel	2000	165	4	2	6	3.64	159	100	94.34	3.7735849	1.89	2.37	[64]
29	Africa	2016	61	2	1	3	4.92	58	103	97.17	1.8867925	0.94	1.90	[65]
30	India	2004	23	0	0	0	0.00	23	106	100.00	0	0.00	0.00	[66]
31	Chinese	2015	16	0	0	0	0.00	16	106	100.00	0	0.00	0.00	[67],
32	Nigeria	2014	41	1	1	2	4.88	39	104	98.11	0.9433962	0.94	1.42	[28]
33	Pakistan	2011	47	4	3	7	14.89	40	99	93.40	3.7735849	2.83	5.30	[68]
34	Karnataka, India	2014	102	0	2	2	1.96	100	104	98.11	0	1.89	0.99	[69]
35	Turkey	2012	1	0	0	0	0.00	1	106	100.00	0	0.00	0.00	[30]
36	India	2012	1	0	0	0	0.00	1	106	100.00	0	0.00	0.00	[70]
37	Turkey	2012	1	1	0	1	100.00	0	105	99.06	0.9433962	0.00	0.96	[71]
38	India	2011	1	0	0	0	0.00	1	106	100.00	0	0.00	0.00	[72],
39	Turkey	2010	1	0	0	0	0.00	1	106	100.00	0	0.00	0.00	[73]
40	Ethiopia	2006	5	1	1	2	40.00	3	104	98.11	0.9433962	0.94	1.90	[11],
41	India	2010	1	0	0	0	0.00	1	106	100.00	0	0.00	0.00	[74]
42	Nigeria	2010	1	0	0	0	0.00	1	106	100.00	0	0.00	0.00	[75]
43	Brazil	2009	12	0	0	0	0.00	12	106	100.00	0	0.00	0.00	[76],
44	India	2007	51	2	1	3	5.88	48	103	97.17	1.8867925	0.94	2.03	[77],
45	Jordan	2007	5	0	1	1	20.00	4	105	99.06	0	0.94	0.93	[78]
46	China	2006	25	0	1	1	4.00	24	105	99.06	0	0.94	0.78	[79]
47	South Africa	2013	45	0	0	0	0.00	45	106	100.00	0	0.00	0.00	[80]
48	Ethiopia	2005	8	0	0	0	0.00	8	106	100.00	0	0.00	0.00	[81],
49	Italy	2004	70	3	3	6	8.57	64	100	94.34	2.8301887	2.83	3.80	[82]
50	Jordan	2003	1	0	0	0	0.00	1	106	100.00	0	0.00	0.00	[83]
											1.3018868	0.62		

#### Chi-square test

The male participants reported more medicinal plants than women, and it could be stated that males possess more knowledge about the use of medicinal plants than women (Additional file 1). The chi-square on the number of species of plants reported by the two age categories showed important differences. Table 4 represents the median for a number of medicinal species reported by the participants 36–46 and > 46 years of age. Scattering of knowledge was observed in different age groups. The significantly higher average number of medicinal plants (*p* < 0.05) were mentioned by participants of 69 to 79 years (37.88) for men and (24.1) for women, respectively. There were no significant variations (χ^2^ = 13.45; *P* > 0.05) in the < 36 year age group. Analysis of variance (*p* = 0.05) was used to elucidate the effect of gender, age, and gender to gender interaction on the traditional knowledge of plants in society.

**Table 4:** Literature on preliminary in vitro screening of most cited plants

S/No	Plant Species	Activity	References
1.	*Anethum graveolens*	Antibacterial and antimicrobial activity	[85, 86]
2.	*Cynodon dactylon*	Antibacterial and wound healing activity	[87, 88]
3.	*Bergenia ciliata*	Antibacterial, antibacterial, anti –inflammatory and antiviral activity	[89, 90]
4.	*Adiantum venustum*	Antibacterial, antifungal and anti-inflammatory activity	[91]
5.	*Gerbera gossypina*	Antimicrobial activity	[92]
6.	*Aconitum chasmanthum*	Antimicrobial activities	[93]
7.	*Trigonella foenum-graecum,*	Anti-inflammatory, antibacterial and antifungal activities	[94]
8.	*Verbascum thapsus,*	Anti-inflammatory, antimicrobial, antiviral, and anti-hyperlipidemic activity	[95]
9.	*Saussurea lappa*	Anti-inflammatory activity	[96]
10.	*Rosa chinensis*,	Antimicrobial activities	[97]
11.	*Gerbera gossypina*	Antimicrobial activities	[98]
12.	*Taxus wallichiana*	Antibacterial and antifungal activites	[99]
13.	*Aquilegia pubiflora*	Antimicrobial activity	[100]
14.	*Salix babylonica*	Anti-bacterial and anti-fungal activities	[101]
15.	*Sonchus asper*	Antimicrobial activities	[102]
16.	*Prunus armeniaca*	Antimicrobial activity	[103]
17.	*Momordica charantia*	Antibacterial and antifungal activity	[104]
18.	*Urtica dioica*	Antibacterial and antifungal activity	[105, 106]
19.	*Dodonaea viscosa*	Antifungal activity	[107]
20.	*Bergenia stracheyi*	Antifungal activity	[108]
21.	*Pisum sativum*	Antifungal activity	[109]
22.	*Butea monosperma*	Antifungal, antibacterial and anti-inflammatory activities	[110]
23.	*Commelina benghalensis*	Anti-inflammatory and wound healing activities	[111]
24.	*Polygonum nepalense*	Antimicrobial. And antifungal activity	[112]
25.	*Valeriana jatamansi*	Anti-inflammatory activity	[113]
26.	*Cannabis sativa*	Antimicrobial activity	[114]
27.	*Plantago major*	Antibacterial activity	[115]
28.	*Berberis lycium*	Antibacterial, antifungal and healing properties	[116]
29.	*Taraxacum officinale*	Antimicrobial activity	[117]
30.	*Myrsine Africana*	Antimicrobial activity	[1]
31.	*Allium sativum*	Antimicrobial and wound Healing	[118]
32.	*Allium cepa*	Antimicrobial activities	[119]
33.	*Pinus roxburgii*	Antibacterial activity	[120]
34.	*Senecio chrysanthemoides*	Antifungal and antibacterial activities	[121]
35.	*Olea europaea*	Antimicrobial activity	[122]
36.	*Isodon rugosus*	Antimicrobial activities	[123]
37.	*Micromeria biflora*	Antimicrobial activities	[124]
38.	*Lawsonia inermis*	Antimicrobial and antibacterial activities	[125, 126]
39.	*Teucrium stocksianum*	Anti-microbial activities	[127]
40.	*Delbergia sissoo*	Anti-microbial activities	[128]

#### Previous literature on phytochemicals, pharmacological activities, and toxicity

A large number of plants stated in this study possess skin cure possessions and might have compound that are indirectly or directly active against parasites. These compounds are known as secondary metabolic compounds. Medicinal plants used for skin diseases were investigated for preliminary in vitro studies, essential phytochemicals and toxicity from the previous studies. Some of the plant species used for skin ailments have been reported for numerous secondary metabolites which show the significance of the plants in traditional remedies (Table 4).

Preliminary in vitro screening of some of the most mentioned plants have been mentioned to validate the findings of the present study (Table 5). In spite of the wide application of active metabolic compounds for humans; they also have a health hazardous effect because of much toxins. These substances not only hamper with the growth of parasite also have lethal effects on mammalian cells (Additional file 1: Table S1). It is, therefore, important to validate the toxic effects of medicinal plant products in relation to their anti-nutritional and other side effects.

**Table 5:** Phytochemical activities and toxicity of medicinal plants used for skin diseases

S/No	Family / Scientific name / coll. #	Phytochemicals	Toxicity
1.	Acanthaceae *Justicia adhatoda* L. LI 58	Alkaloids, phenolic, flavonoids and sterols [129]	Less toxicity [130]
2.	Amaryllidaceae *Allium cepa* L. LI 6	Alkaloids, flavonoids, cardiac glycosides, terpene, steroids and resins [131]	None
3.	Amaryllidaceae *Allium sativum* L LI 7	Saponin, steroids, tannins, carbohydrates and cardiac glycosides [132]	Excessive use cause toxicity like acute toxicity, burning sensation in the mouth and gastrointestinal tract, nausea, diarrhea, vomiting [133]
4.	Apiaceae *Anethum graveolens* L. LI 10	Essential oils, fatty oil, proteins, carbohydrates, fiber and ash [134]	Nontoxic [135]
5.	Apiaceae *Coriandrum sativum* L*.* LI 33	Alkaloids, carbohydrates, volatile oil, tannins, and flavonoids [136]	Acute and sub chronic toxicity [137]
6.	Apiaceae *Ferula foetida* (Bunge) Regel. LI 47	Terpenoids, Sulfide derivatives, volatile Oil and Phenols [138]	Little toxicity including (including lung metastasis) [139]
7.	Apiaceae *Pleurospermum brunonis* Benth. ex C.B.Clarke LI 80	None	None
8.	Apocynaceae *Calotropis procera* (Aiton) Dryand. LI 24	Cardenolides, flavonoids, and saponins [132] .	Highly toxic [140]
9.	Apocynaceae *Carissa spinarum* L. Haines LI 22	Alkaloids, tannin, glycoside, steroids and carbohydrates [141]	Acute toxicity (Shamim, 2014)
10.	Apocynaceae Rauvolfia serpentina L. LI 84	Phenolic acids and flavonoids [142]	None
11.	Asteraceae *Anaphalis margaritacea* (L.) Benth. & Hook.f. LI8	Flavonoids, polyacetylenes, and hydroxylactone [143]	
12.	Asteraceae *Artemisia vulgaris* L. LI 12	Carbohydrate, saponins, phytosterol, proteins, amino acid, tannin & phenolic compounds and flavonoids [144]	Genotoxicity [145]
13.	Asteraceae *Gerbera gossypina* (Royle) Beauverd LI 50	None	Less toxicity [139]
14.	Asteraceae *Gnaphalium affine* D.Don LI 51	Flavonoids, sesquiterpenes, diterpenes, Triterpenes and phytosterols [146]	Damage oxidative compounds and produce various toxic compound that are harmful for humans [139]
15.	Asteraceae *Launaea nudicaulis* (L.) Hook.f. LI 60/	Flavonoids, anthocynadins and flavanones [147]	Nontoxic [148]
16.	Asteraceae *Saussurea lappa* (Decne.) Sch.Bip. LI 93	Alkaloids, glycosides, phenolics, steroids and terpenoids [149]	Acute toxicity [150]
17.	Asteraceae *Senecio chrysanthemoides* DC LI 94	Triterpene, emodins,polyphenol, reducing sugar and anthocyanosides [151]	Hepatotoxicity [150]
18.	Asteraceae *Sonchus asper* (L.) Hill LI 96	Ascorbic acid, carotenoids and fatty acids [152]	Acute toxicity [153]
19.	Asteraceae *Taraxacum officinale* aggr. F.H. Wigg. LI 99	phenolic compounds, flavonoid glycosides [154]	Acute toxicity [155]
20.	Asteraceae *Tussilago farfara* L. LI 103	Terpenes, flavonoids, and alkaloids [156]	Acute toxicity [157]
21.	Balsaminaceae *Impatien edgeworthii* Hook. f LI 54	Flavonoids, sugars, alkaloids and saponins [158]	Cytotoxicity [159]
22.	Berberidaceae *Berberis lycium* Royle LI 15	ß-sitosterol, 4,4-dimethylhexadeca-3-ol, Butyl-3-hydroxypropyl phthalate, Butyl-3-hydroxypropyl phthalate and 4-methyl-7-hydroxycoumarin [160]	Acute toxicity and oral toxicity [158]
23.	Boraginaceae *Hackelia americana* (A.Gray) Fernald LI 52	Phenols, saponins, and flavonoids [161]	Hepatotoxicity [162]
24.	Boraginaceae *Onosma hispida* Wall. ex G. LI 71	Flavonoid, amines, iridoids and sesquiterpene [163]	Acute toxicity [164]
25.	Brassicaceae *Brassica juncea* (L.) Czern. LI 20	2,6-dichlorophenol indophenol and HEPES 4-(2-Hydroxyethyl)-1- piperazine-ethane-sulphonic acid [165]	Poisonous [166]
26.	Buxaceae *Buxus papillosa* C.K. Schneid. LI 21	Cyclobuxupaline-C (IV)(+)-cyclopapilosine-D (VII) and (+)-buxamine-C [167]	Nonpoisonous [168]
27.	Cannabaceae *Cannabis sativa* L LI 26	Alkaloids, flavonoids, cardiac glycosides, resins, terpins and steroids [169].	High doses cause inhibition of hepatic drug and decreased fertilization capacity [170]
28.	Capparaceae *Capparis decidua* (Forssk.) Edgew. LI 27	alkaloids, phenols, sterols and glycosides [171]	Acute toxicity [172]
29.	Caprifoliaceae *Valeriana jatamansi* Jones ex Roxb. LI 105	Phenols, flavonoids and tannins [173]	Fumigant toxicity [174]
30.	Caryophyllaceae *Cerastium fontanum* subsp. *vulgare* (Hartm.) Greuter & Burdet, LI 29	None	None
31.	Commelinaceae *Commelina benghalensis* L LI 32	Terpenoids, saponins, tannins, flavonoids, steroids, phenolic compounds, alkaloids and cardiac glycosides [175]	Acute and sub-acute toxicity, male reproductive toxicity [176]
32.	Convolvulaceae *Cuscta reflexa* Roxb. LI 35	Flavonoids and tannins [177]	Oral toxicity [178]
33.	Cucurbitaceae *Cucumis melo* L. LI 36	Alkaloids, terpenoids, carbohydrate, proteins, flavonoids, phytosterols [179]	Metal toxicity [180]
34.	Cucurbitaceae *Lagenaria siceraria* (Molina) Standl. LI 59	Protein, carbohydrates, Flavonoid and saponin [181]	Gastrointestinal toxicity [182]
35.	Cucurbitaceae *Momordica charantia* L. LI 67	Alkaloid, glycoside, aglycone, tannin, sterol, phenol, protein and carbohydrate [183]	Hepatotoxicity [184]
36.	Cupressaceae *Juniperus communis* L*.* LI 56	Steroids, alkaloids, phenolics, flavonoids, tannins and terpenoids [185]	Nephrotoxicity [186]
37.	Cupressaceae *Juniperus excelsa* M. Bieb. LI 57	Alkaloids,flavonoids, phenols, saponins and diterpenes [187]	Cytotoxicity [188]
38.	Cyperaceae *Cyperus difformis* L LI 38	Flavonoids, coumarins, tannins and sterols [189]	Fumigent toxicité [190](Chang et al., 2012)
39.	Elaeagnacea*e Hippophae rhamnoides* L. LI 53	Phenol, Quercetin and Catechin [191]	Non toxic [192]
40.	Equisetaceae *Equisetum arvense* L. LI 43	Flavonoids, alkaloids, minerals, phenolic petrosins, triterpenoids, saponins, phytosterols [193]	Acute and metal toxicity [194]
41.	Euphorbiaceae *Euphorbia helioscopia* L. LI 44	Reducing sugars, terpenoids, alkaloids, steroids, tannins, flavanoids and phenolic compounds [195]	Cytotoxicity [196]
42.	Fabaceae *Butea monosperma* (Lam.) Kuntze LI 14	Sterols, triterpenes, glycosides flavonoids and proteins [197].	Acute and oral toxicity [198]
43.	Fabaceae *Delbergia sissoo* L. LI 41	Proteins, phyto sterols, tannins, starch, flavonoids and tannins [199].	Acute toxicity [200]
44.	Fabaceae *Pisum sativum* L. LI 77	Tannins, terpenoides, alkaloids and flavonoids [201]	Cadmium toxicity in human [202]
45.	Fabaceae *Trigonella foenum-graecum* L LI 102	Alkaloids, cardiac glycosides, and phenols [203]	Acute toxicity [204]
46.	Gentianaceae *Swertia abyssinica* Hochst. LI 97	None	Hepatic toxicity [205]
47.	Lamiaceae Ajuga integrifolia Buch-Ham-ex D. Don LI 5	Essential oil [206]	Body weakness [205]
48.	Lamiaceae *Isodon rugosus* (Wall. ex Benth.) LI 55	Alkaloids, glycosides, flavonoids, oils, terpenoids, saponins, tannins and anthraquinones [207]	Cytotoxicity [159]
49.	Lamiaceae *Micromeria biflora* (Buch.-Ham. ex D.Don) Benth LI 66	None	Membrane toxicity of cell [184]
50.	Lamiaceae *Nepeta hindostana* (B.Heyne ex Roth) Haines. LI 68	None	Mycotoxin [208]
51.	Lamiaceae *Rydingia limbata* (Benth.) Scheen & V.A. Albert LI 90	None	Cytotoxicity [209]
52.	Lamiaceae *Salvia moorcroftiana* wall. ex Benth LI 92	Flavonoids, diterpenoids and sterols [210]	Nontoxic inhibitor [211]
53.	Lamiaceae *Teucrium stocksianum* Boiss. LI 101	Alkaloids, tannins, flavonoids, saponins, steroid, reducing sugar, terpenoid, anthraquinone, phlobatannin and glycoside [212]	Acute toxicity [213]
54.	Loranthaceae *Loranthus pulverulentus* Wall LI 62	Triterpenoids, alkaloids, carbohydrates, flavanoids, proteins, tannins and glycosides [214]	Low toxicity [148]
55.	Lythraceae *Lawsonia inermis* L. LI 61	Glycosides, phytosterol, steroids, saponins, and tannins [215]	Highly toxic [148]
56.	Malvaceae *Abelmoschus esculentus* (L.) Moench LI 1	Carbohydrate, gums and mucilages, proteins, phytosterols, flavonoids, tannins, phenolic Compounds and volatile oil (Saha et al., 2011).	No toxic effect [216]
57.	Meliaceae *Melia azadarach* L. LI 65	Alkaloids, Tannins, Saponins, Phenols [217]	Toxic [218]
58.	Myrsinaceae *Myrsine africana* L. LI 63/	Saponins, tannins, flavonoids, amino acids, steroids and reducing sugar [219]	Acute toxicity [148]
59.	Nitrariaceae *Peganum harmala* L. LI 72	Alkaloids, flavonoids and anthraquinones [220]	Cytotoxicity [221]
60.	Nyctaginaceae *Boerrehavia diffusa* L. LI 19/	1,1-diphenyl picrylhydrazyl, phenolic, flavonoid and ascorbic acid [222]	Acute toxicity [223]
61.	Oleaceae *Olea europaea* subsp. *cuspidata* (Wall. & G.Don) Cif LI 70	Flavonoids, terpenes [224]	Low toxicity [164]
62.	Papaveraceae *Corydalis govaniana* Wall. LI 34	Alkaloids [225]	Acute toxicity (Mukhopadhyay et al., 1987)
63.	Phytolaceae *Phytolacea letsenia* L. LI 73		None
64.	Pinaceae *Cedrus deodara* (Roxb. ex D.Don). LI 28	Tannins, flavanoids, alkaloids, and terpenoids [226]	Cytotoxicity [172]
65.	Pinaceae *Pinus roxburgii* Sarg LI 75/	Flavonoids and terpenoids [227]	Acute toxicity [228]
66.	Pinaceae *Pinus wallichiana* A.B. Jacks. LI 76	Flavonoid and phenolic [229]	Toxic [228]
67.	Plantaginaceae *Picrorhiza kurrooa* Royle. ex Benth. LI 74	Sterols, glycosides and phenolic compounds [230]	Cytotoxicity [231]
68.	Plantaginaceae *Plantago major* L*.* LI 78	Alkaloids, flavonoids, saponins, quinones, terpenes, lignans, tannins, polysaccharides, steroidal glycoside, thiosulfinates, proanthocyanidin and proteins [232]	Less toxicity [233]
69.	Plantaginaceae *Plantago lanceolata* L. LI 79	Anthraquinone, Glycosides and alkaloids [234]	Not toxic [235]
70.	Poaceae *Cynodon dactylon* (L.) Pers. LI 37	Alkaloids, anthroquinone, flavonoids, saponins, steriods, tannins and triterpenoid [190]	Fungal growth, biomass toxicity [236]
71.	Polygonaceae *Fagopyrum acutatum* (Lehm.) Mansf. ex K.Hammer LI 45	Protein, carbohydrates, fat and rutin [237]	Hepatotoxicity [238]
72.	Polygonaceae *Polygonum nepalense* Meissn. LI 81	None	Toxic [239]
73.	Polygonaceae *Rumex abyssinicus* Jacq. LI 87	Tannins, anthraquinones, amino acids flavonoids and carbohydrates [240]	Non toxic in cell [241]
74.	Polygonaceae *Rumex dissectus* H. Lév. LI 88	B-carotene linoleic acid, has antioxidant activity [242]	Less toxic [243]
75.	Polygonaceae *Rumex dentatus* L. LI 89	Alkaloids, terpenoids, flavonoids and tannins [244]	Toxic [174]
76.	Polygonaceae *Fagopyrum tataricum* (L.) Gaertn. LI 46	Flavonoids [245]	Cytotoxicity [246]
77.	Primulaceae *Androsace rotundifolia* Lehm. ex Roem. & Schult. LI 9	None	Less toxic [247]
78.	Pteridaceae *Adiantum venustum* D. Don LI 4	Adininaneone, adininaonol and Norhopan [248]	Nontoxic (Huxley et al., 1992)
79.	Ranunculaceae *Aconitum chasmanthum* Stapf ex Holmes LI 2	Alkaloids, benzoylmecasonine and mesaconitine [249]	Some species are highly poisonous [250]
80.	Ranunculaceae *Aconitum delphinifolium* DC. LI 3	Alkaloids, benzoylmecasonine and mesaconitine [249]	Slightly poisonous when used in access [250]
81.	Ranunculaceae *Aquilegia pubiflora* Wall. ex Royle LI 13	None	Nontoxic [251]
82.	Ranunculaceae *Caltha alba* Cambess LI 25**/**	Alkaloides, flavonoids, glycosides and triterpenoides [252]	Acute toxicity, cytotoxicity [216]
83.	Ranunculaceae *Nigella sativa* L. LI 69	Flavonoid glycosides quercetin and kaempferol 3-glucosyl [253]	Hepatotoxicity [254]
84.	Rhamnaceae *Colubrina oppositifolia* Brongn. ex H. Mann LI 23		None
85.	Rosaceae *Malus pumila* Mill. LI 64	Triterpenoids and flavonoids [255]	Hepatotoxic [148]
86.	Rosaceae *Prunus armeniaca* L. LI 82	Carbohydrates, phenolic compounds and organic acids [256]	Acute and renal toxicity [257]
87.	Rosaceae *Prunus persica* (L.) Batsch LI 83	Phenolics, anthocyanins and flavonoids [258]	Toxic side effects [259]
88.	Rosaceae *Rosa chinensis* Jacq LI 85	None	None
89.	Rosaceae *Rubus abchaziensis* Sudre LI 86	Diterpene glycosides, phenolic glycoside and Lignan glycoside [260]	Cytotoxicity and mitochondrial toxicity [261]
90.	Rubiaceae *Galium abaujense* Borbás LI 48	None	None
91.	Rubiaceae *Gallium aparine* L*.* LI 49	None	None
92.	Rutaceae *Zanthoxylum armatum* DC LI 11	Limonene,linalool,neral [262]	Cytotoxic and Phytotoxic potential [263]
93.	Rutaceae *Citrus medica* L. LI 30	Carbohydrates, proteins, amino acids and flavonoids [264]	Estrogenic effect [265]
94.	Rutaceae *Citrus sinensis* L. LI 31	tannin, alkaloid, saponin, flavonoid, steroid, tripertenes [266]	Fumigant toxicity [267]
95.	Salicaceae *Salix babylonica* L. LI 91	Phenolics and saponins [268]	Cytotoxicity [269]
96.	Sapindaceae *Dodonaea viscosa* (L.) Jacq LI 42	Carbohydrates, flavonoids, proteins, amino acids, saponins, steroids, sterols, tannins, and triterpenoids [270]	Acute toxicity [271]
97.	Saxifragaceae *Bergenia ciliata* (Haw.) Sternb LI 16	Alkaloids, carbohydrates, cardiac glycosides, saponins, phenols, flavonoids and diterpenes [272].	Acute toxicity [273]
98.	Saxifragaceae *Bergenia ligulata* Engl. LI 17	Bergenin, catechin, gallicin and gallic acid [274]	Radical toxicity in renal epithelial cell [275]
99.	Saxifragaceae *Bergenia stracheyi* Hook.f. & Thomson) Engl LI 18	Bergenin 2. Tannic acid 3. Gallic acid 4. Stigmesterol 5. β-Sitosterol 6. catechin 7 [276]	Acute toxicity [277]
100.	Scrophulariaceae *Verbascum thapsus* L. LI 106	Methanolic extract has antiviral activity against the pseudorabies virus [278]	Toxic pyrrolizidine alkaloids [279]
101.	Solanaceae *Datura stramonium* L. LI 40	Saponins, tannins, alkaloids and glycosides [280]	Poison and hallucinogen [281]
102.	Solanaceae *Solanum virginianum* L. LI 95	None	Cytotoxicity [282]
103.	Tamaricaceae *Tamarix aphylla* (L.) H. Karst. LI 98	Flavonoids, alkaloids and tannins [283]	Less toxic [284]
104.	Taxaceae *Taxus wallichiana* Zucc. LI 100	Diterpenoids, lignans, flavonoids, steroids and sugar derivatives [285]	Hepatotoxicity [286]
105.	Thymelaeaceae *Daphne mucronata S* Royle LI 39	Coumarins, flavonoids, triterpenoids, lignin, glucosides, daphnine and umbelliferone [287]	Leaf extract is highly toxic [287]
106.	Urticaceae *Urtica dioica* L. LI 104	Phytosterols, saponins, flavanoids, tannins, hydrolysable tannins, phenolic compounds, proteins and amino acids [288]	Nontoxic [289]

#### Comparison with other studies in neighbouring regions

In the present study, some plants were used alone to treat the particular diseases, while in some cases plant parts were mixed to treat diseases. This present study reported 63 novel plants for skin diseases from Northern Pakistan, including *Ajuga integrifolia*, *Anaphalis chitralensis*, *Capparis himalayensis, Gnaphalium affine, Isodon rugosus, Tamarix aphylla, Nepeta clarkei*, *Launaea nudicaulis*, *Valeriana jatamansi* (Table 2).

## Discussion

This study was carried out in the native groups of Northern Pakistan. People use medications for the cure of several diseases. Generally the medicinal plants are used in village parts of the area. The majority of professional healers in this study were males, this finding is similar to the literature [290]. According to an estimate, 84% of the rural population relies on herbal traditional medicinal plants [291]. Different origins of the medicinal plant knowledge were recorded. The inherited knowledge of medicinal plants is transferred through orally a cultural practice common in the rural areas in addition to the divine revelation. Most people inherit traditional knowledge from their elders that passed generation to generation [292].

The most dominant life form uses in the study was herbs. Herbs are easily available and collected from roadsides and farmlands [293–295]. Asteraceae was the most preferred family used. Previous work [3] also reported Asteraceae (6 species), Lamiaceae (6 species) and Fabaceae (5 species) with large figure of medicinal flora. There seems to be a tendency for a few families of plants to stand out in any pharmacopeia [296]. These plant families have been reported with high pharmacological, organoleptic and pharmaceutical properties [297]. The fewer species were observed in 37 families that are similar to previous studies [298, 299].

Among the reported plant part leaves were the most used plant part. In various studies, leaves were reported to be used as powder and paste on the affected skin areas [300]. The powder was found to be the most preferred method of utilization. The use of powder and decoction is the major mode of utilization in the herbal preparations in the ethnomedicinal studies by [35, 301]. The preparations were applied 2–3 times daily until healing occurred. A large number of herbal preparation involved soaking the plant material in water for a few days and taking the infusion, while few involved boiling the parts of plants and take the decoction. The drugs were usually prepared from the paste of the plant part either with water, lime water, rose water, coconut water, milk, ghee, and butter. Sometimes juice extract from fresh parts of plants was used. Treatments were done with single plant parts or a combination of different parts of the same plant. The amount of powder used to make a concoction was defined as a half, full or a quarter of a teaspoon. In the morning, the mixtures were regularly used before breakfast or afterward dinner, for 3-7 successive days, or till the patient was completely cured.

The medicinal plants described in this study for the cure of skin infections might also be utilized additionally for their phytochemical and pharmacological activities. Following reports carried out in various areas also described the common practices of medicinal species usage against the diseases of skin [22, 29, 48].

The overall effectiveness of the mentioned plant species in the context of curing skin ailments was calculated on the basis of the computed index called used value [40]. This species was mentioned by 21 participants. Wounds and skin burns treated by *Pisum sativum* showed an increase in oxygen supply as a result of increased blood pressure flow [302]. In other studies glycoprotein extracted from *Pisum* helped the formation of epidermis tissues [303]. The highest UV for important medicinal plants like *Pisum sativum* and *Cynodon dactylon* might be ascribed to the trends of using herbal drugs for skin diseases in the area. It is also observed that plant species that are using repeatedly are more possibly to be active biologically and have good healing properties [53]. Less available in the study site parallel to small UV e-g in case of *Adiantum venustum* [304].

Relative frequency of citation is applied to choose high potential medicinal plant species for future research anti-skin diseases drug development. The medicinal species that have high RFC should be further analyzed for phytochemical compounds, to recognize their active chemical components for drug discovery [305]. These findings might be considered as of greatest importance for relating and assessing study in associated hypothetical fields for upcoming drug inventory and sustainable utilization of plant species for medicinal purposes [306].

The plant species that were cited only once by a single participant were not considered for the fidelity level study. The high value of FL indicates the choice of participants to treat the specific disease [84]. These plants can be verified as significant medicinal flora on additional estimation by the help of pharmaceutical, phytochemical and biological actions. We have found the species as more significant having 80 FL% or greater.

In [292] the maximum value of FIV was documented for Juglandaceae (45%) followed by Punicaceae (44%) whereas the lowest value was noted for Vitaceae and Rubiaceae (3%) The results of present study vary from previous literature reports due to differences in climate and vegetation of area [307]. The highest percentage of FIV demonstrates that the plants of a particular family are commonly used in curing many diseases as reported by participants.

Jaccard index is used to find out the similarity of medicinal uses with previous studies carried out on skin ailments. The maximum level of resemblance was present in findings carried out in North-West Frontier Province, Pakistan and Gilgit Baltistan Pakistan on skin diseases [1, 73]) with Jaccard index value 11.81 and 5.30, respectively. About 12% average similarity is reported among different areas and the study regions. The recent study represents a high level of novelty index with respect to the use of medicinal species in skin diseases and its significance in old traditional recipes [308] specified in his study work that the medicinal plants repeatedly cited must be utilized as herbal drug development. The comparison of similarities shows the significant authenticity of documented data. Similarly, the medicinal plants which are not cited in previous work should be assessed for pharmacological and phytochemical analysis for drug discovery development.

In this research, the use of medicinal plants against skin diseases were studied for the occurrence of various toxicity and phtochemicals stated in former literature (see Table 5, Additional file 1). Mostly all the species had been described previously for their one or more phytochemical important compound representing their importance in medicinal cures. In the study, phytochemical analysis on genus, Aconitum has directed to the identification of alkaloids, benzoyl mecasonine and mesaconitine [249]. Some species of *Aconitum* are slightly poisonous when used in the excess amount [250]. In other studies, *Bergenia ciliate* was reported to contain active compounds such as alkaloids, carbohydrates, cardiac glycosides, saponins, phenols, flavonoids and diterpenes [272]. *Allium sativum* is rich with saponins, steroids, tannins, carbohydrates, allicin and cardiac glycosides which possess essential skin diseases curing activity [132]. Alkaloids, flavonoids, phenols, saponins and diterpenes compounds of *Juniperus excels* also have reported skin properties [185]. High consumption of flavonoids and phenolics may inhibit enzyme activity and cause oxidative damage [309]. Some alkaloids can inhibit enzyme activity, block ion channels loss of coordination, convulsions, hallucination and even death [310]. Myrsine Africana reported to have an acute toxic effect and *Malus pumila* cause hepatotoxicity [148], *Rubus fruticosus* damage cell activity that was stated by [261]. Discovery of drugs from medicinal plants links a multidisciplinary approach to joining pharmacological, botanical, ethnomedicinal and natural methods. Some natural products of plant derivatives are in the phase of the trial and are in experimental use [311]. Therefore further pharmacological, ethnomedicinal and phytochemical studies should be carried out to authenticate the use of plant species in skin diseases and to discover new drugs.

The root of *Butea monosperma* was reported for skin diseases in the present study while it is reported as a blood purifier and skin diseases in the work of [312]. *Coriandrum sativum* was used to control hypertension, joint pain, stomach complaints, and Gastrointestinal tracts problems [313], but in the present study, it is reported to treat pimples and skin problems. Fruits of *Lagenaria siceraria* were reported to treat severe body pain [314], while our study revealed that fruits and seeds can be used for skin problems. The leaves of *Justicia adhatoda* have been used for muscular pains in a study of [315], but this study documented that the leaves can be used for wound healing. Leaves of *Myrsine africana* were reported for stomach problems in the previous studies of [313], these results are in accord with the present study. The flowers and leaves of *Verbascum thapsus* were used for wounds [314], while the current study found that aerial parts of plant’s may be utilized for the cure of blemishes and several skin related problems.

*Launaea nudicaulis* and *Gnaphalium affine* were used often for skin ailments. Asteraceae are generally rich in flavonoids, sesquiterpenes, diterpenes, triterpenes, phytosterols [146]. *Nepeta clarkei, Ajuga integrifolia,* and *Isodon rugosus* were used for curing of boils, wound healing and skin problems, respectively. *Capparis himalayensis* was used for wound healing in areas of Northern Pakistan. The medicinal use of species related to wound healing was not reported earlier. *Euphorbia helioscopia* was reported for the treatment of cholera, jaundice, respiratory diseases, cancer [46], but the present study reported it for wound healing. *Brassica juncea* was found to treat some skin problems while the literature suggested it for the treatment of ulcer*s* [316]. In this study, *Cucumis melo* was used to treat skin burn while in a previous study it was used to treat liver diseases [314]. This study showed that *Rheum emodii* can be used for skin ailments, while in literature it is mostly reported for the treatment of cancer [317]. Our research also found that *Swertia alata*, as used for skin diseases, while the previous study reported it only as used for rheumatic disorders [314]. *Onosma hispida* was documented to treat skin burns, compared to use as skin tonic [318]. *Verbascum thapsus* also served for curing skin ailments, while traditionally it was reported for stomach diseases [319]. *Melia azedarach* was found as a treatment for pimples and wound healing, but literature reported this species for sexual problems and as skin tonics [320]. The present work therefore suggest that public sector administrator in study area should make policies in order to protect people from health problems and use of medicinal plants by local people for treatment of diseases.

## Conclusions

This is the first quantitative ethnomedicinal study that provides information about the use of 106 species that belonging to 90 genera and 56 families for the treatment of skin diseases in Northern Pakistan. Key findings of the study revealed leaves to be the most used plant parts (58%), herb to be dominant life form (63%) and powder to be the most frequent method of administration (22%). The highest skin disease category was recorded for wound healing (40%). RFC ranged from 0.07 to 0.25%, highest use-value reported for *Pisum sativum* (0.143 UV), highest FIV was observed for Pteridaceae (26.6 FIV) while FL values ranged from 100% to 36.8. The medicinal information documented in this study could be explored in the future for phytochemical and pharmacological investigations which may lead to plant-based nano-medicine drug discovery and development.

## Additional file

Additional file 1: **Table S1.** Chi-square test χ^2^ test for gender wise distribution. **Figure S1.** Schematic representation of medicinal plant parts used prepared by NVivo software for skin diseases in Northern Pakistan. **Figure S2.** Systematic representation of mode of utilization for skin diseases in Northern Pakistan. (DOCX 615 kb)

## Data Availability

Not Applicable.

## Abbreviations

FC: Frequency of citation
FL: Fidelity Level
IBC: Institutional Bio-ethics Committee
ISL: Islamabad
JI: Jaccard index
Pak: Pakistan
RFC: Relative Frequency of citation
THPs: Traditional Health Practitioners
Qau: Quaid-i-Azam uni
