# Supplementary material for: An ethnobotanical study of medicinal plants used to treat skin diseases in northern Pakistan
Source: BMC Complement Altern Med. 2019 Aug 13;19:210. doi: 10.1186/s12906-019-2605-6 (PMC6693210; doi:10.1186/s12906-019-2605-6)
Supplement: Supplementary file 1 — Table S1. Chi-square test χ2 test for gender wise distribution. Figure S1. Schematic representation of medicinal plant parts used prepared by NVivo software for skin diseases in Northern Pakistan. Figure S2. Systematic representation of mode of utilization for skin diseases in Northern Pakistan. (DOCX 615 kb) [file 12906_2019_2605_MOESM1_ESM.docx]

**Table 6** Chi-square test ᵪ^2^ test for gender wise distribution

| **Observed frequencies of Gender** | | | |
| --- | --- | --- | --- |
| **Age group** | **Female** | **Male** | **Total** |
| **36-46 Years** | 08 | 12 | 20 |
| **47-57 Years** | 18 | 17 | 35 |
| **58-68 Years** | 18 | 25 | 43 |
| **69-79 Years** | 17 | 45 | 62 |
| **80> Years** | 09 | 11 | 20 |
| **Total** | **70** | **110** | **180** |

| **Expected frequencies of gender** | | | |
| --- | --- | --- | --- |
| **Age group** | **Female** | **Male** | **Total** |
| **36-46 Years** | 7.7 | 12.2 | 20 |
| **47-57 Years** | 13.6 | 21.3 | 35 |
| **58-68 Years** | 16.7 | 26.2 | 43 |
| **69-79 Years** | 24.1 | 37.8 | 62 |
| **80> Years** | 7.7 | 12.2 | 20 |
| **Total** | **70** | **110** | **180** |

| **Computation of value of ᵪ^2^** | | | | |
| --- | --- | --- | --- | --- |
| **o** | **E** | **(o-e)** | **(o-e)^2^** | **ᵪ^2^=(o-e)^2^/e** |
| **08** | 7.7 | 0.3 | 0.09 | 0.011 |
| **18** | 13.6 | 4.4 | 19.36 | 1.423 |
| **18** | 16.7 | 1.3 | 1.69 | 0.101 |
| **17** | 24.1 | -7.1 | 50.41 | 2.09 |
| **09** | 7.7 | 1.3 | 1.69 | 0.21 |
| **20** | 12.2 | 7.8 | 60.84 | 4.98 |
| **35** | 21.3 | 13.7 | 187.69 | 8.81 |
| **43** | 26.2 | 16.8 | 282.24 | 10.77 |
| **62** | 37.8 | 24.2 | 585.64 | 15.49 |
| **20** | 12.2 | 7.8 | 60.84 | 4.98 |
| **180** | **247** |  |  | **48.865** |

**Critical region**:

The degree of freedom= (n_1)= 5-1=4; The value of **ᵪ^2^** _(0.05, 4)=_ 9.49, The critical region is **ᵪ^2^** >9.49

**
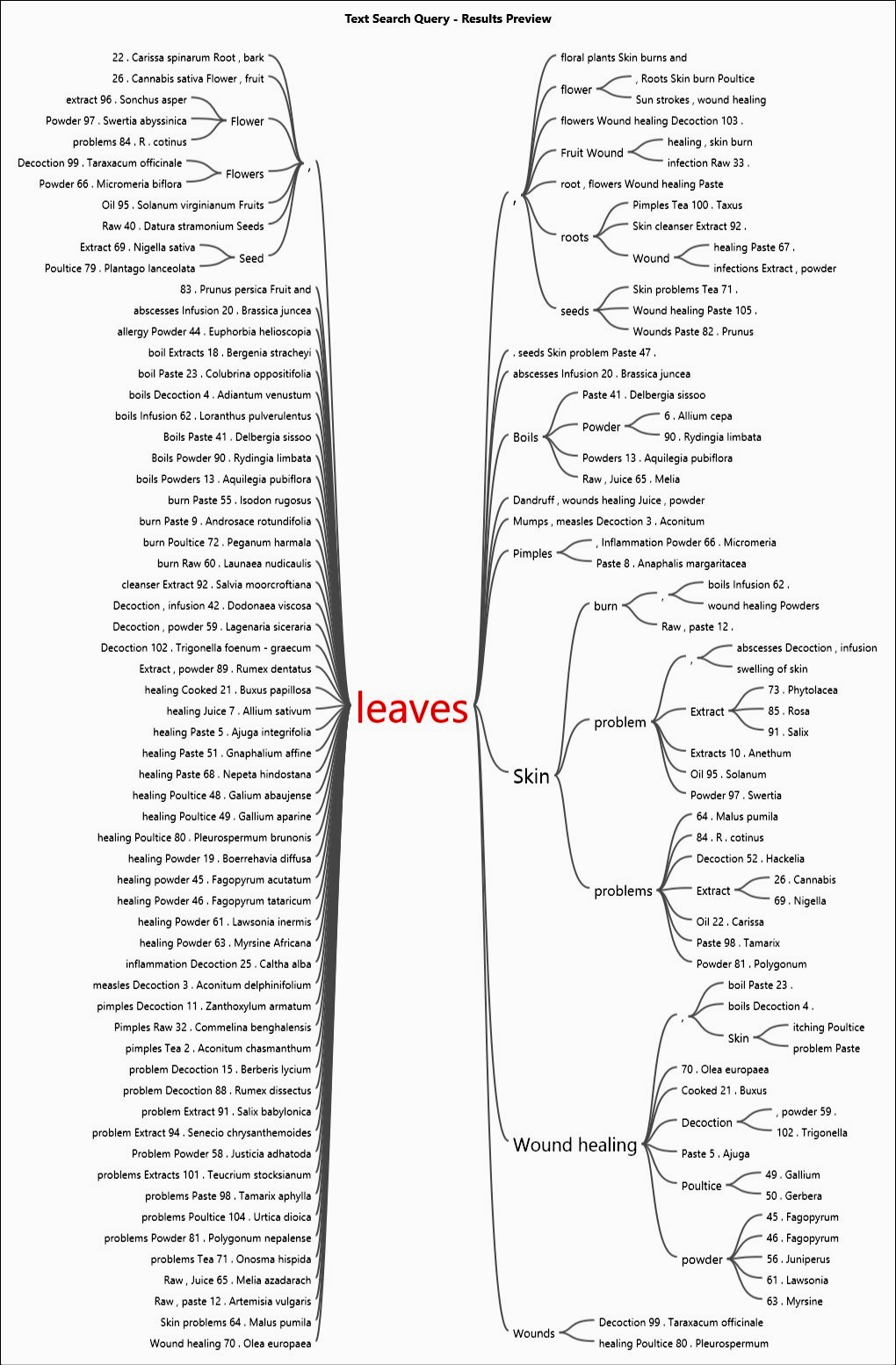
**

**Fig. 5** Schematic representation of medicinal plant parts used prepared by NVivo software for skin diseases in Northern Pakistan


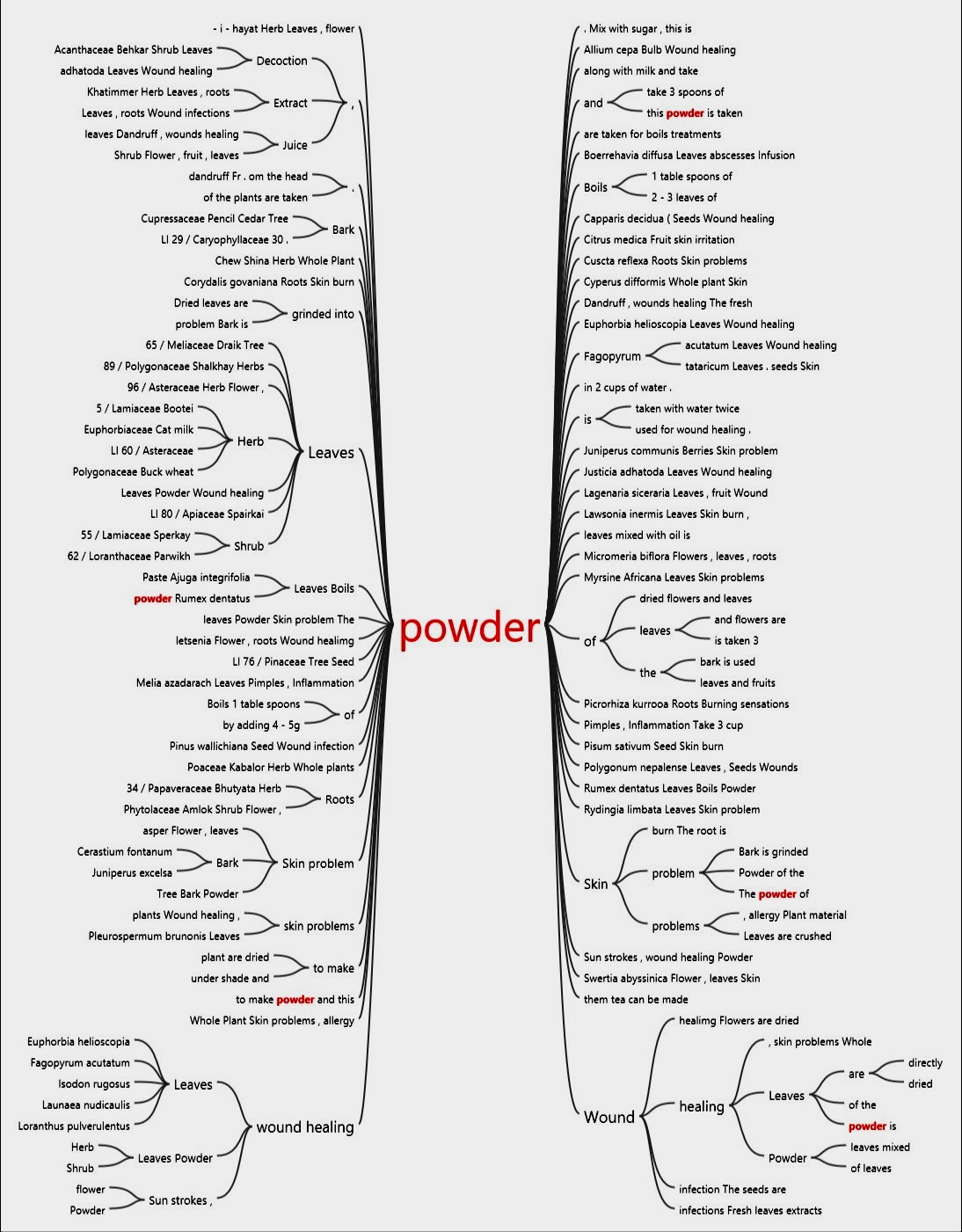


**Figure 7** Systematic representation of mode of utilization for skin diseases in Northern Pakistan
